# A phage-derived reconfigurable effector associated with an actinobacterial contractile nanomachine tailors bacterial responses to competition

**DOI:** 10.1128/jb.00532-25

**Published:** 2026-05-06

**Authors:** Toshiki Nagakubo, Tatsuya Nishiyama, Shumpei Asamizu, Hiroyasu Onaka, Nobuhiko Nomura, Masanori Toyofuku

**Affiliations:** 1 Institute of Life and Environmental Sciencesm, University of Tsukuba 13121, Tsukuba, Japan; 2 Tsukuba Institute for Advanced Research (TIAR), University of Tsukuba 13121, Tsukuba, Japan; 3 Microbiology Research Center for Sustainability (MiCS), University of Tsukuba 13121, Tsukuba, Japan; 4 Life Science Research Center, Nihon University 622935 , Fujisawa, Japan; 5 Engineering Biology Research Center, Kobe University 12885, Kobe, Japan; 6 Graduate School of Agricultural and Life Sciences, The University of Tokyo 13143, Bunkyo, Japan; 7 Faculty of Science, Gakushuin University 155344, Toshima, Japan; 8 Life Science Center for Survival Dynamics, University of Tsukuba 13121, Tsukuba, Japan; Southern University of Science and Technology, Guangdong, China

**Keywords:** *Streptomyces*, contractile injection system, Actinobacteria, microbial interaction, phage, tape measure protein, effector functions

## Abstract

Contractile injection systems (CISs) are derivatives of phage tails and are widely distributed in prokaryotes. CISs load cognate effectors and eject them through contractile actions resembling those of phage tails. Ejected effectors play central roles in CIS functionality by acting on target cells and mediating various biological processes. Here, we report a novel group of CIS effectors related to phage tape measure protein, the transmembrane component of the phage infection machinery. This group is broadly distributed within the class Actinobacteria, one of the bacterial classes in which CIS gene clusters are highly conserved, and is represented by Sle1, a cognate effector of the intracellularly localized *Streptomyces lividans* phage tail-like nanoparticle (SLP). This effector is associated with Sle2, which contains a CIS effector core domain and interacts with the SLP core component. Sle1 is packaged inside SLP and is translocated to lipid membranes along with SLPs. The functional domain of Sle1 enriches the membrane-associated subproteome in *S. lividans* and *E. coli*. This effect modifies the physiological properties of *S. lividans*, ultimately enhancing its adaptation to microbial competition. In addition, we revealed that Sle1-type effectors conserved among actinobacterial species are structurally and functionally diverse in their functional domains. One of them from *Micromonospora eburnea* constitutes a novel toxin-antitoxin system, and introducing its functional domain into Sle1 reprograms the phenotypic responsiveness of *S. lividans* to neighboring bacteria. Our findings illustrate that phage elements can be incorporated into CISs as reconfigurable platforms for bacterial adaptation to various environmental conditions.

**IMPORTANCE:**

Bacterial CISs have attracted interest for their importance in microbial ecology and potential in biotechnological applications. However, understanding of their functional diversity is currently limited because many CIS effectors remain unannotated due to a lack of inferable structural and genetic signatures. Our findings on Sle1 and its relatives illuminate a previously unidentified class of CIS effectors with phage tape measure protein-related modular architecture, association with the CIS effector core domain, and wide distribution within the major class of Actinobacteria, substantially expanding the known repertoire of effector classes. The impact of Sle1 on *S. lividans* suggests a link between CIS effectors and bacterial adaptation to environmental conditions, highlighting unexplored functional diversity of CIS effectors as tuners of bacterial phenotypes in communities.

## INTRODUCTION

Bacteria and phages, viruses that infect bacterial cells, are involved in a continuous arms race. Although recent studies have characterized various systems that protect bacterial cells from phage infection and its detrimental ecological consequences by eliminating phage elements, bacteria often maintain genes encoding phage tail-like nanostructures within their genomes (1–4). Such nanostructures are no longer infectious and can be ecologically beneficial to the host bacteria under certain circumstances (5, 6).

Contractile injection systems (CISs) are a group of prokaryotic phage tail-like nanostructures with macromolecular structures and mechanisms of action resembling those of the contractile tails of myophages. Both CISs and myophages are composed of a central, hollow tube encased by a sheath that contracts during action, with a baseplate complex connecting them to the spike. On the basis of the conservation of structural characteristics, CISs have been proposed to have evolutionarily diverged from tailed phages (7), perhaps through evolutionary events that caused genetic immobilization. While phages inject nucleic acids into target cells through tail contraction, CISs are not associated with viral genetic material but instead load certain effectors inside the tube lumen and eject them through contractile action similar to that of phages. The ejected effectors can act on target cells through their enzymatic activities or other unknown mechanisms (8, 9). Because of their sophisticated structures and functional versatility, CISs have been employed by a wide range of prokaryotic species as mediators of various biological processes (8–10). At present, known CISs and CIS-related nanostructures can be grouped into two classes based on their localization. Some CISs are extracellularly released, after which effectors are injected into target cells by attaching their fibers to the cell surfaces (8–11). Others are intracellularly localized, and several studies have implied their potential evolutionary relationship with type VI secretion systems, a class of gram-negative bacteria-specific phage tail-like nanomachines (12). Although the former class of extracellular CISs has been relatively well-investigated and characterized, the latter class of intracellular CISs remains poorly understood, particularly in terms of their cognate effectors. Given the rapidly increasing interest in the medical and biotechnological applications of CISs through the modification of effectors (13, 14), a comprehensive understanding of the natural effector repertoire is required.

*Streptomyces* has long been investigated as a producer of various bioactive metabolites, including antibiotics, and is notable for its highly conserved CIS-related gene clusters (15). In our previous studies, we identified and characterized a *Streptomyces lividans* phage tail-like nanoparticle (SLP) that is closely related to CISs and was originally identified in *Streptomyces lividans* TK23 (16, 17). SLP represents the most widely conserved class of actinobacterial CIS-related nanostructures, including the CIS*^Sc^* of *S. coelicolor*, which is a close relative of *S. lividans* (18). The SLP gene cluster is located in the genetic region adjacent to threonine tRNA, implying that these CIS-related nanostructures are derivatives of an ancient phage infecting bacteria using tRNA loci as attachment sites (19). In the *Streptomyces* life cycle, which encompasses the vegetative growth of substrate mycelia and subsequent aerial mycelial erection and spore formation, SLPs are mainly produced during the vegetative growth (17). SLPs are unlikely to target other organisms, as they are localized in the cytoplasm of *S. lividans* throughout its life cycle and lack typical tail fiber components (16, 17). In addition, SLPs are potentially associated with a complex protein-protein interaction network involving ribosomal proteins, and their involvement in microbial interactions has been suggested (16, 17). Although these observations suggest a potential role for SLPs in the central cellular systems of the producer bacterium, the cognate SLP effector and its function remain unclear.

Here, we report the identification of a cognate effector of SLP and its effect on the producer bacterium. We show that the cognate SLP effector represents a novel class of CIS effectors and that its functional domain broadly affects the cellular functions of *S. lividans* by modulating cellular protein profiles. We also propose that the functionality of these effectors was acquired and diversified from the phage infection machinery to foster the adaptation of producer bacteria to varying environmental conditions.

## RESULTS

### Identification of the SLP cargo

**Fig 1:**
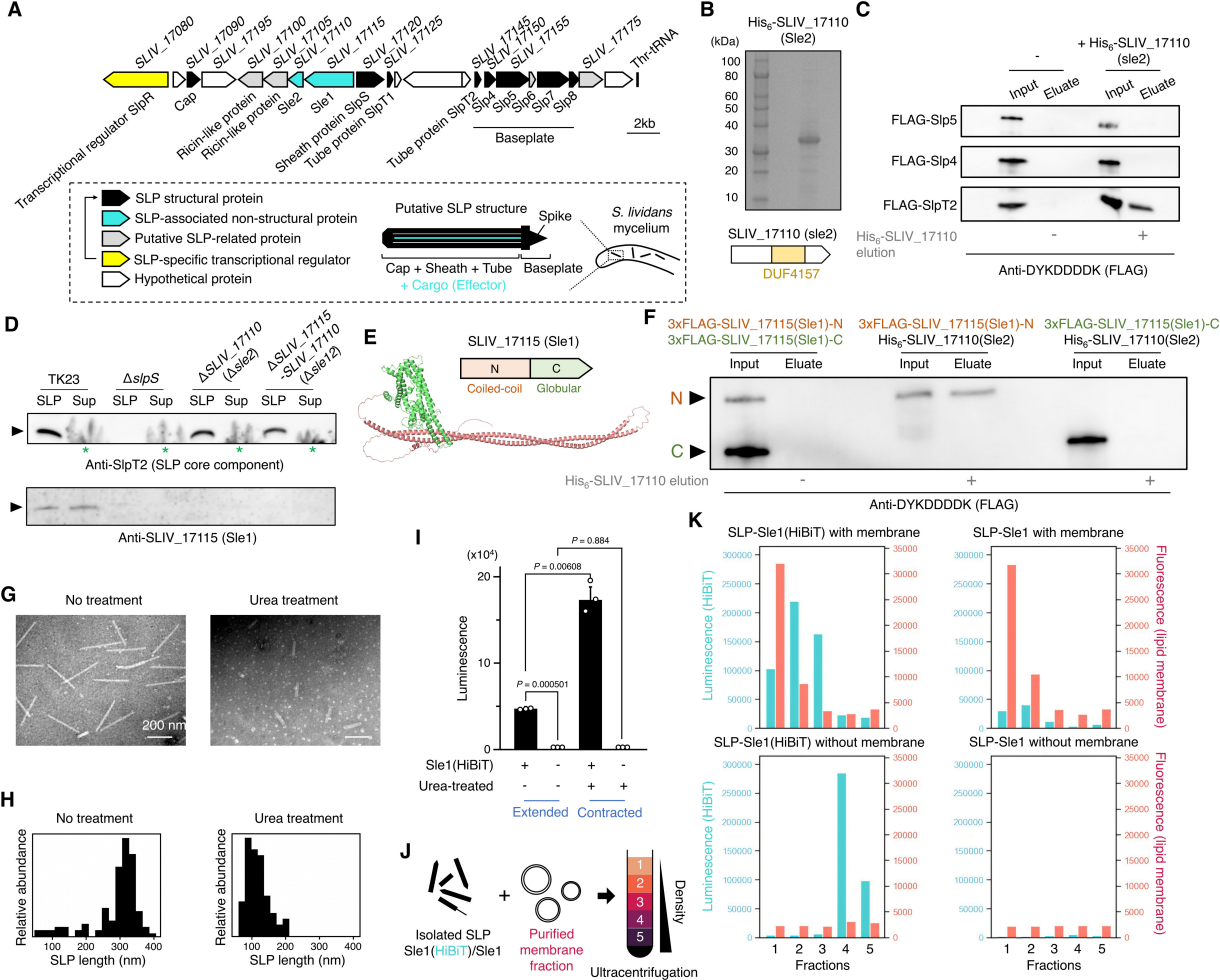
SLP loads a cargo protein SLIV_17115 (Sle1) that can be translocated to lipid membranes. An SLP cargo protein associated with a CIS core domain-containing protein was identified. (**A**) SLP gene cluster is shown. SLP structural proteins were identified based on proteome analysis and western blotting of the isolated SLPs. SlpR is a transcriptional regulator specific to the SLP gene cluster. (**B**) SLIV_17110 (Sle2) containing the CIS core domain (DUF4157 domain) was cloned and purified as a hexahistidine (His_6_)-tagged protein. (**C**) DYKDDDDK (FLAG)-tagged SLP structural proteins SlpT2, Slp4, and Slp5 were mixed individually with the purified His_6_-SLIV_17110 (Sle2) and then subjected to Ni^2+^-affinity chromatography. The inputs and eluates were analyzed by western blotting using an anti-DYKDDDDK antibody. (**D**) Substrate mycelia of *S. lividans* grown on a solid medium were scraped off the plate and separated into SLP fractions (SLP; ultracentrifugation pellet) and detergent-soluble fractions (sup; ultracentrifugation supernatant). These fractions were further separated by SDS–PAGE and then subjected to western blotting. Green asterisks indicate that the signal of SlpT2 was not clearly detected in the sup fractions due to the presence of a detergent, which would have interfered with the migration of SlpT2. (**E and F**) The structure of SLIV_17115 (Sle1) was predicted using AlphaFold3, and the predicted coiled-coil N-terminal and globular C-terminal regions were subjected to a co-elution assay with His_6_-SLIV_17115 (Sle1). DYKDHDGDYKDHDIDYKDDDDK (3 × FLAG) was fused to the N-termini of the SLIV_17115 (Sle1) fragments. The tagged proteins were mixed individually with His_6_-SLIV_17110 (Sle2) and subjected to Ni^2+^-affinity chromatography. Western blotting was performed using an anti-DYKDDDDK antibody. (**G**) The isolated SLPs were treated with 3 M urea or buffer and then observed by transmission electron microscopy after dilution to an appropriate concentration. (**H**) Length distributions of the images in *F* were analyzed. Distributions around 100 and 300 nm indicate the contracted and extended SLPs, respectively. (**I**) SLPs complemented with either Sle1(HiBiT) or the native one were isolated and treated with 3 M urea or buffer. After dilution to an appropriate concentration, the treated SLPs were subjected to a HiBiT-LgBiT split luciferase assay. Bars indicate mean ± S.D. for three independent assays. *P* values were calculated by *t*-test with Welch’s correction. (**J**) Experimental design for an interaction assay between SLP and lipid membranes. (**K**) Density-gradient ultracentrifugation samples were fractionated and then subjected to the HiBiT-LgBiT split luciferase assay and FM1-43 staining. Luminescence and fluorescence indicate the presence of Sle1(HiBiT) and lipid membranes, respectively.

In our previous analysis to identify SLP-associated proteins (17), we noticed that several proteins with unknown functions were identified as being associated with SLPs (Fig. S1). Among these, the hypothetical proteins SLIV_17115 and SLIV_17110 were tandemly encoded just upstream of the SLP structural proteins in the reverse direction (Fig. 1A; Fig. S1). SLIV_17110 has a DUF4157 domain, which has been proposed to be the core domain of numerous CIS effector-related proteins (Fig. 1B) (20). Since interactions between a DUF4157 domain-containing protein and the CIS spike complex were suggested in a previous study on another *Streptomyces* CIS (21), we investigated the interaction between SLIV_17110 and predicted spike complex proteins SlpT2 (tube initiator; Afp5/Pvc5 homolog), Slp4 (spike hub; Afp7/Pvc7 homolog), and Slp5 (spike; Afp8/Pvc8 homolog) (Fig. 1A). His_6_-SLIV_17110 and FLAG-tagged spike complex protein were subjected to coelution assay using Ni^2+^-affinity chromatography, and potential interaction between SLIV_17110 and SlpT2 was detected (Fig. 1B and C). The association between SlpT2 and SLP was confirmed by western blotting (Fig. 1D; Fig. S2).

SLIV_17115, which is encoded just upstream of SLIV_17110, does not share close homology with any of the characterized proteins. This protein was predicted to consist of an N-terminal large coiled-coil segment and C-terminal alpha helices folded into a globular structure (Fig. 1E; Fig. S3 and S4). Two of the C-terminal helices were predicted to form a transmembrane helical hairpin (Fig. S4). SLIV_17115 was predicted to have remote homology with the tape measure protein of Chivirus chi (Fig. S5) (22). Tape measure proteins are phage cargo proteins ejected from the tail tube lumen and have recently been proposed to be the evolutionary origin of some CIS effectors (21). Additionally, gene adjacency to DUF4157 domain-containing proteins has been recently proposed to be a characteristic feature of some CIS effectors lacking the DUF4157 domain (23). Since these observations imply the potential significance of SLIV_17115 as an effector associated with the CIS core domain-containing protein SLIV_17110, we performed an interaction assay for these proteins and found the interaction between SLIV_17110 and the N-terminal region of SLIV_17115 (Fig. 1F). Furthermore, we identified 62 close SLIV_17115 homologs among 754 *Streptomyces* species with publicly available RefSeq genome sequences. Notably, all the SLIV_17115 homologs with the N-terminal extension were flanked by small DUF4157 domain-containing proteins and SlpS (sheath) homologs (Fig. S6 and S7). Therefore, SLIV_17110 would be the conserved partner of SLIV_17115, and SLIV_17110 potentially mediates an indirect connection between SLIV_17115 and the baseplate core of SLP. Since the above results consistently suggested that these proteins are candidate SLP effectors, we named them SLP-associated effector 1 (Sle1) and Sle2, respectively.

We also performed a markerless in-frame deletion of *sle1* and *sle2*. Although Δ*sle2* and Δ*sle1/2* mutants were obtained, our attempt to delete *sle1* alone failed. We speculate that higher-order DNA structure around *sle1* might have affected recombination efficacy around this locus. SLPs isolated from the Δ*Sle1/2* mutant did not show any apparent structural defects (Fig. S8). However, the abundance of Sle1 was dramatically decreased by the deletion of *slpS*, which encodes a sheath protein essential for SLP construction, and *sle2*, suggesting that mature SLP and Sle2 may enhance the biological stability of Sle1 (Fig. 1D; Supplemental notes).

### Sle1 is externalized after urea treatment and can be associated with lipid membrane fractions

To gain insights into the biological functions of the newly identified SLP-associated proteins, we first evaluated their effects on *E. coli* cells. We found that the expression of Sle1 markedly inhibited colony formation in *E. coli*, whereas Sle2 had no detectable effect, suggesting that Sle1 may be a cognate, biologically active effector of SLP (Fig. S9). To investigate whether the contractile action of SLP externalizes Sle1 (11, 24), we explored the conditions that artificially triggered SLP contraction and found that exposure to urea efficiently induced SLP contraction, similar to the typical contractile phage tails (Fig. 1G and H) (25). We next introduced either Sle1-Sle2 or Sle1(HiBiT)-Sle2, with the latter construct including an 11-amino acid peptide tag (HiBiT tag) at the C-terminus of Sle1, into the Δ*sle1/2* mutant. The HiBiT tag activates a 19 kDa inactive luciferase fragment, LgBiT (26) (Fig. S10). Upon the addition of LgBiT and a luminescent substrate (furimazine), significantly stronger luminescence was detected in the SLP fraction of the Sle1 (HiBiT)-Sle2-complemented strain (Fig. 1I). Notably, the luminescence of the SLP fraction of the Sle1(HiBiT)-Sle2-complemented strain markedly increased after exposure to urea, which induced SLP contraction (Fig. 1G through I). This result indicates that the conformational change in SLP during urea-induced contraction externalized Sle1(HiBiT), allowing more LgBiT to access the activator HiBiT tag in Sle1(HiBiT). Sle1(HiBiT) might be ejected from SLP through contraction, or passively exposed as a structural component.

Given that potential interactions between cellular membrane and SLP (CIS*^Sc^*), the close relative of SLP in *S. coelicolor* (18), we investigated whether SLP and Sle1 can be associated with the membranes. Upon incubation of purified lipid membranes of the SLP-deficient (Δ*slpS*) mutant (16) and the isolated SLP containing Sle1(HiBiT), both the effector and SlpS migrated to the lipid membrane fractions (Fig. 1J and K; Fig. S11). This result suggests that Sle1 can be associated with lipid membrane fractions along with SLPs.

### The C-terminal domain of Sle1 interacts with a ribosome-associated fraction and partially and transiently suppresses the *E. coli* growth

**Fig 2:**
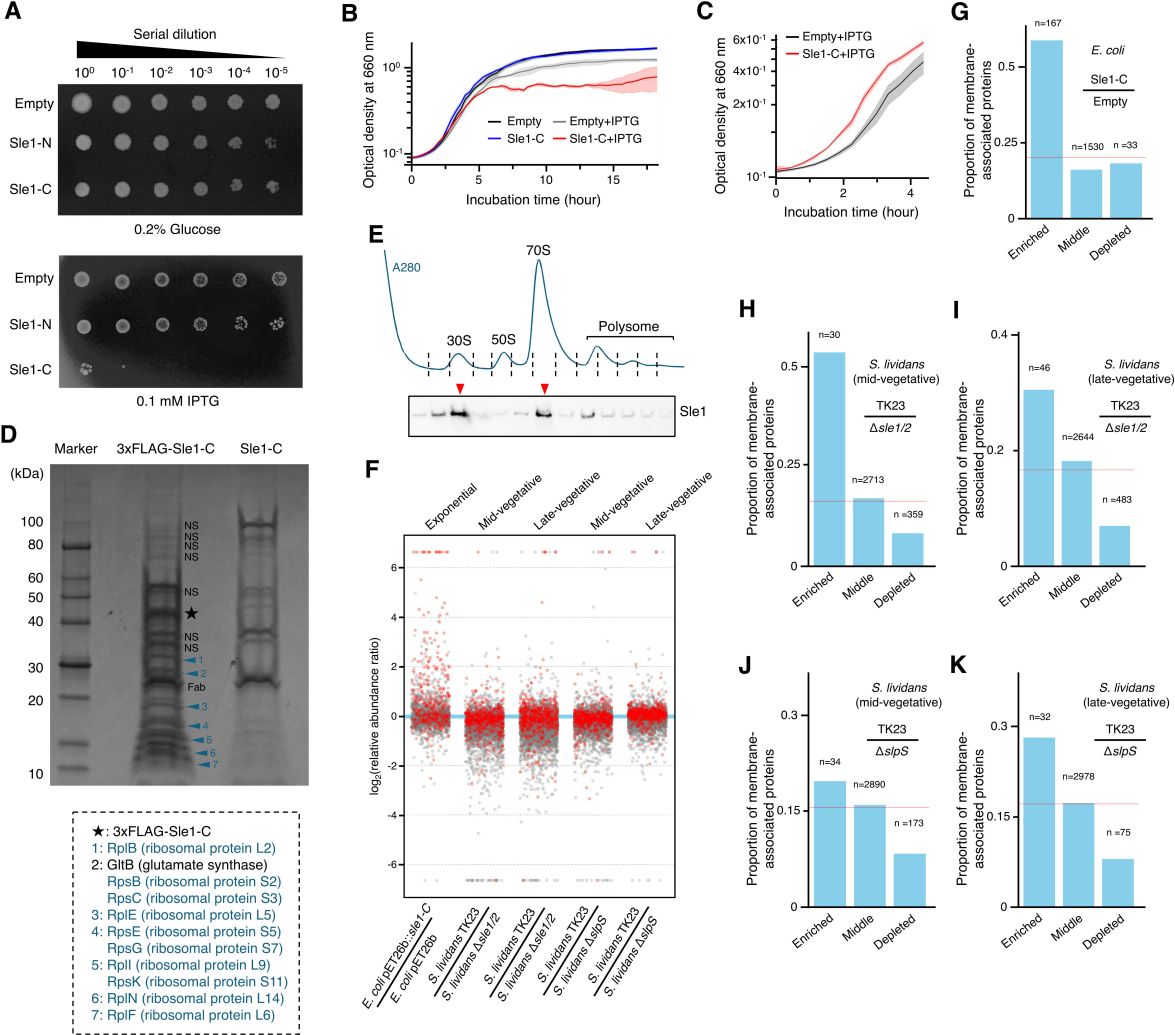
Characterization of Sle1 as a SLP effector impacting bacterial proteome allocation. Sle1 was characterized as an SLP effector that impacts protein profiles in both *E. coli* and *S. lividans*. (**A**) Cultures of *E. coli* NiCo21(DE3) harboring pET26b, pET26b::*sle1-N*, or pET26b::*sle1-C* were serially diluted and spotted onto solid LB media containing a suppressor (0.2% [wt/vol] glucose) or inducer (IPTG, 0.1 mM). (**B**) Growth curves of *E. coli* NiCo21(DE3) harboring pET26b or pET26b::*sle1-C* are shown. 0.025 mM IPTG was added as an inducer. Lines and colored areas indicate the mean values for three independent cultures and S.D., respectively. (**C**) The *E. coli* strains used in panel B were grown to the stationary phase in the presence of IPTG, and then these cells were regrown after diluting to the same optical density at 660 nm with a fresh liquid LB medium. Lines and colored areas indicate the mean values for three independent cultures and S.D., respectively. (**D**) *E. coli* NiCo21(DE3) harboring pET26b::*sle1-C* or pET26b::*3 × FLAG-sle1-C* was grown to the mid-exponential phase in the presence of the inducer, and then 3 × FLAG-Sle1-C was pulled down with anti-DYKDDDDK antibody fragment (Fab)-conjugated agarose beads. Potential interacting proteins were identified using MALDI-TOF-MS and MASCOT search. NS, non-specific band. (**E**) Ribosomes were extracted from the substrate mycelia of *S. lividans* and purified by sucrose density-gradient ultracentrifugation. Each fraction was subjected to western blotting analysis using anti-Sle1 serum. (**F**) Total proteins were extracted from the indicated bacterial strains and subjected to quantitative proteomic analysis. The abundance ratio refers to the abundance of each of the detected proteins (scatter plot) in a reference strain (numerator) relative to that in a control strain (denominator). Proteins with inferred localization in the membranes are displayed in red. The upper and lower limits for the calculation of the abundance ratio were 100- and −100-fold, respectively. (**G–K**) Proportions of the predicted membrane proteins detected by the proteome analysis in panel F were calculated and displayed as bars. Proteins were classified as “enriched” or “depleted” if their abundances in the numerator strains were >2 or <−2-fold, respectively, compared to those in the denominator strains. The remaining proteins were classified as “middle.” Red lines indicate mean proportions of the predicted membrane proteins in total proteins commonly detected in two strains compared. Membrane localization of the detected proteins was predicted based on UniProtKB, which combines experimental data in Swiss-Prot and sequence-based computational prediction.

To determine the Sle1 domain responsible for the inhibition of colony formation in *E. coli*, we assayed the truncated N- and C-terminal regions of Sle1. Only the C-terminal region (Sle1-C) exhibited an inhibitory effect on *E. coli* cells, indicating that this is a functional domain (Fig. 2A). Because some effectors associated with phage tail-like nanomachines have been proposed to have bactericidal activities (20), we investigated whether Sle1-C kills *E. coli* by measuring the growth of the *E. coli* strains in liquid medium with or without induction of Sle1-C. Notably, Sle1-C expression resulted in partial suppression of growth, and Sle1-C-expressing *E. coli* cells in the stationary phase were resuscitated immediately after inoculating into fresh medium without the inducer (Fig. 2B and C). Therefore, Sle1-C is likely to cause transient and partial suppression in *E. coli* growth rather than killing the bacterium through bactericidal activity.

We could not find any significant structural motifs with known enzymatic functions in Sle1-C, leading to the hypothesis that Sle1-C partially suppresses *E. coli* growth by a mechanism other than enzymatic disruption of biomolecules. Based on this hypothesis, we performed a co-immunoprecipitation assay to identify Sle1-C targets in *E. coli*. The 3 × FLAG-tagged Sle1-C or the non-tagged version was heterologously expressed in *E. coli,* and the cell lysates were mixed individually with agarose beads conjugated with anti-DYKDDDDK (FLAG tag) antibody fragments. Subsequent mass spectrometry analysis identified most of the proteins associated with the immunoprecipitated Sle1-C as ribosomal proteins constituting the 30S and 50S subunits of the *E. coli* ribosome (Fig. 2D). Given the potential association of heterologously expressed Sle1-C with ribosomal proteins, we also analyzed the ribosome fractions isolated from *S. lividans* mycelia and found that Sle1 was mainly detected in the 30S and 70S ribosome fractions, indicating an association between Sle1 and the subcellular fractions containing the small ribosomal subunit in *S. lividans* (Fig. 2E).

### Membrane proteins are enriched among proteins increased in abundance upon Sle1-C expression

Since Sle1-C did not inhibit the activity of the protein synthesis system in *E. coli* (Fig. S12; Supplemental notes), we assumed that Sle1-C might indirectly alter the protein expression profiles of *E. coli* cells by interacting with the ribosome-associated fraction rather than inhibiting ribosomal activity. To test this hypothesis, we performed quantitative proteome analysis of the *E. coli* strains harboring either the Sle1-C expressing plasmid or the empty plasmid. Overall, at the mid- to late stage of the exponential growth phase, Sle1-C-expressing cells were more abundant in experimentally or bioinformatically identified membrane proteins than control cells (Fig. 2F and G; Supplemental data). Statistically significant correlations between the abundance ratios relative to the control sample and the annotations as membrane proteins were only detected in the two proteome subsets with abundance ratios of ≥2.0 (167 proteins; point-biserial correlation coefficient, 0.243; *P* value, 0.00158) or ≥1.0 and <2.0 (698 proteins; point-biserial correlation coefficient, 0.0865; *P* value, 0.0222). The proteome subset with an abundance ratio of ≥2.0 included membrane proteins that play roles in respiration (NADH-ubiquinone oxidoreductase subunits A, N, H, and L), saccharide uptake (PTS system EII components), peptidoglycan synthesis (MurJ), and protein export (SecD/F/Y, tatE). Thus, it is possible that over-accumulation of these membrane proteins may cause an imbalance of metabolic fluxes, limiting the ability of cells to adapt to varying nutritional conditions and consequently leading to slower growth. Furthermore, western blot analysis using purified lipid membranes from *E. coli* indicated that Sle1-C was partially localized to the membranes, consistent with the relevance of Sle1-C to membrane proteins (Fig. S13). Sle1-C might have interacted with the membranes through hydrophobic interactions between the predicted transmembrane helices and lipid layers.

Given the significant changes in the *E. coli* proteome upon Sle1-C expression, we further performed proteome analysis of the *S. lividans* strains TK23, Δ*slpS*, and Δ*sle1/2*. The results revealed an overall downshift in the relative protein abundance ratios in the parental TK23 strain compared to the deletion mutants during the mid- to late vegetative growth phase (Fig. 2F). No consistent enrichment of stress response-related proteins, such as major molecular chaperones and heat shock proteins, was observed (Fig. S14; Supplemental data). In contrast, we also found that experimentally or bioinformatically identified membrane proteins were consistently abundant in the proteome subsets where the relative abundance ratios of proteins were significantly higher (>2-fold) in the parental TK23 strain than in either of the deletion mutants. The opposite trend was observed in the subsets where the abundance ratios were lower in the TK23 strain (Fig. 2F, H through K; Supplemental data). We did not observe molecular species-specific changes in protein abundance in the above proteome subsets across strains or time points.

### The Sle1/2 pair tunes the metabolic activity of *S. lividans* to maintain reproductive activity under competition

**Fig 3:**
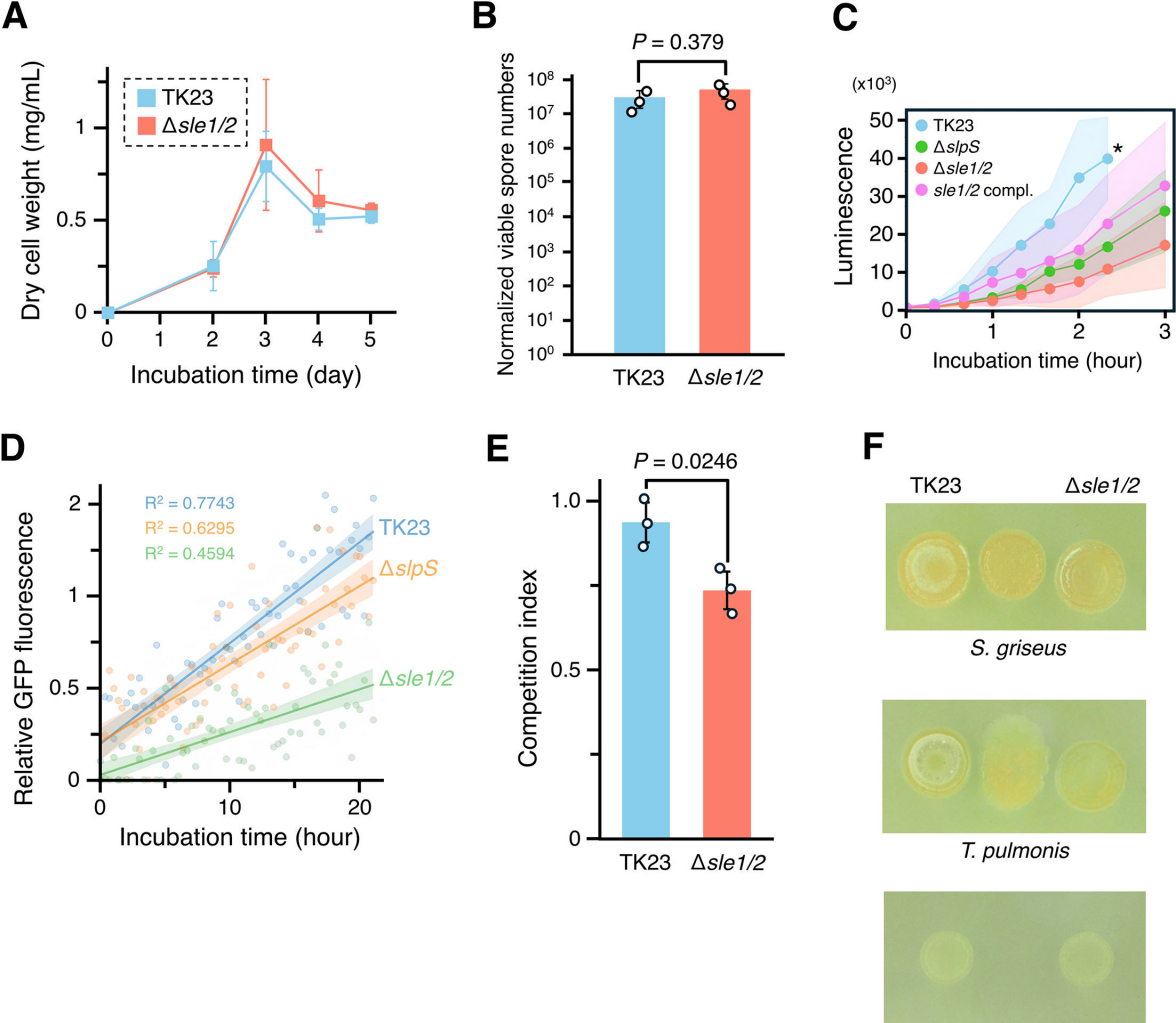
The Sle12 pair contributes to the maintenance of reproductive activity of *S. lividans* under competition. The Sle12 pair potentially confers an ecological benefit to *S. lividans* by enhancing adaptability under competition for nutrients. (**A and B**) Vegetative growth and spore formation were compared between the parental TK23 strain and the Δ*sle12* mutant by measuring dry cell weight in liquid media and the number of viable spores formed on solid media, respectively. Values and error bars indicate means ± S.D. for five (**A**) or three (**B**) independent cultures. (**C**) Cellular reducing activity of each *S. lividans* strain was measured using a cell-permeable luminescence substrate and luciferase as described under the “Materials and Methods” section. Values and colored areas indicate means ± S.D. for three independent cultures. The asterisk indicates the time point after which the luminescence of “TK23” exceeded the detection threshold. (**D**) Protein synthesis capacity of *S. lividans* strains expressing msfGFP was measured. In these constructs, msfGFP expression was regulated by an engineered *tipA* promoter (P*_tipA_*RS), in which transcription and translation of the downstream open reading frames were dependent on exogenously added thiostrepton and theophylline, respectively. Relative GFP fluorescence was calculated by subtracting the fluorescence of mycelia under non-inducing conditions from that under inducing conditions. The *S. lividans* strains in this panel are shown as the genetic backgrounds of the msfGFP-expressing strains. Scatter plots, lines, and colored areas indicate the mean values for three independent samples, regression lines, and 95% confidence intervals, respectively. (**E**) *S. lividans* strains harboring a thiostrepton resistance gene and *Streptomyces griseus* were inoculated on a solid sporulating medium, and then spores were collected from the co-culture. The competition index was calculated as the viable spore number of *S. lividans* relative to that of *S. griseus*. The *S. lividans* strain in this panel is shown as the genetic background of the thiostrepton-resistant strain used for the competition assay. (**F**) *S. lividans* strains were co-cultured with *S. griseus* or *Tsukamurella pulmonis* on solid media without direct contact for 3 days. The white regions of the colonies indicate aerial mycelia.

Given the broad impact of Sle1 on the *S. lividans* proteome, we were interested in how this was reflected in the phenotypes of this bacterium. We measured the vegetative growth of the parental TK23 strain and the Δ*sle1/2* mutant and obtained similar growth curves (Fig. 3A). Spore formation following the vegetative growth of substrate mycelia in the *Streptomyces* life cycle (27) was also comparable between these strains (Fig. 3B). We next investigated energy metabolism of the *S. lividans* strains, since Sle1-associated enrichment of energy metabolism-related membrane proteins was observed (Fig. 2F through K; Supplemental data). We applied an assay system based on luciferase and a cell-permeable substrate for monitoring cellular reducing activity, as cellular membrane permeability of the *S. lividans* strains appeared comparable (Fig. S15). We found that substrate mycelia of the Δ*sle1/2* mutant showed lower reducing activity than the parental TK23 strain and the *sle1/2*-complemented strain (Δ*sle1/2 attC::sle1/2*) at the mid- to late stage of vegetative growth, indicating that loss of the Sle1/2 pair would lead to lower energy metabolism at this stage (Fig. 3C). We also examined the capacity of the substrate mycelia for energy-consuming protein synthesis by monitoring inducible msfGFP expression in *S. lividans* strains. Notably, the linear regression slope of msfGFP fluorescence of the Δ*sle1/2* mutant was lower than that of the TK23 strain at the mid- to late stage of vegetative growth, which is consistent with the lower energy metabolism of the mutant (28) (Fig. 3D). In addition, the Δ*slpS* mutant also showed lower cellular reducing activity and msfGFP induction than the parental TK23 strain at the same growth stage (Fig. 3C and D). Lower abundance of *sle1* could have limited Sle1 function and led to partial decreases in the metabolic activity in this mutant. Furthermore, at the early stage of vegetative growth, the Δ*sle1/2* mutant showed higher cellular reducing activity and msfGFP induction (Fig. S16).

Since the decrease in energy metabolism and protein synthesis in the Δ*sle1/2* mutant at the later growth stage suggests the vulnerability of this mutant to a nutrient-limited condition, we tested various growth conditions under which *S. lividans* potentially experiences low-nutrient stress. We found that the spore formation of this strain was relatively lower than that of the parental strain in co-culture with *Streptomyces griseus*, which competes with *S. lividans* for nutrients (Fig. 3E; Fig. S17). In addition, we observed an apparent delay in aerial mycelial erection in the Δ*sle1/2* mutant colony co-cultured with the competitor without direct contact (Fig. 3F). A delay in mycelial erection was also observed in the mutant colony in a co-culture with *Tsukamurella pulmonis*, which has been characterized as intimately interacting with *Streptomyces* species in nature and not producing antibiotics significantly effective against *S. lividans* (29–31) (Fig. 3F).

### A wide distribution of Sle1/2-like proteins in the major actinobacterial class and their functional diversity

**Fig 4:**
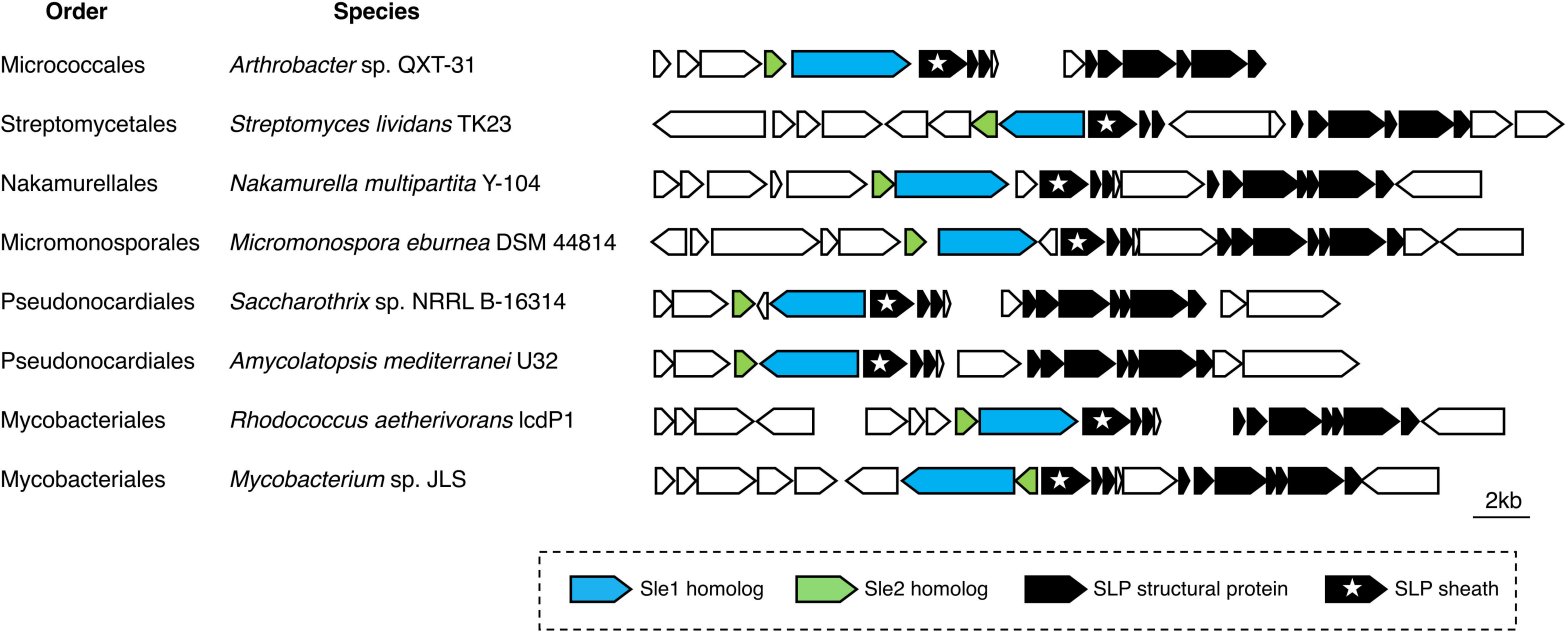
Distribution of Sle1/2-like pairs in the major actinobacterial class. Homologs of Sle1 and Sle2 are widely conserved in the class Actinobacteria with consistent gene synteny.

Given the unique function of Sle1, we were interested in whether Sle1 and its partner Sle2 are conserved among bacteria. We first searched for Sle2 homologs using the eCIStem database (20) and found that they were located within various CIS-related gene clusters conserved in the phylum Actinobacteria (Fig. 4). Notably, they were frequently encoded adjacent to alpha helix-rich proteins and just upstream of CIS sheath protein homologs. The alpha helix-rich proteins include large coiled-coil segments at the N-termini and share remote homology with phage tape measure proteins (Fig. S18). All these structural features and genomic contexts are consistent with those of Sle1, suggesting that Sle1/2-like pairs are widely shared across Actinobacteria, probably serving as cognate effectors for CIS-related nanomachines beyond *Streptomyces*.

**Fig 5:**
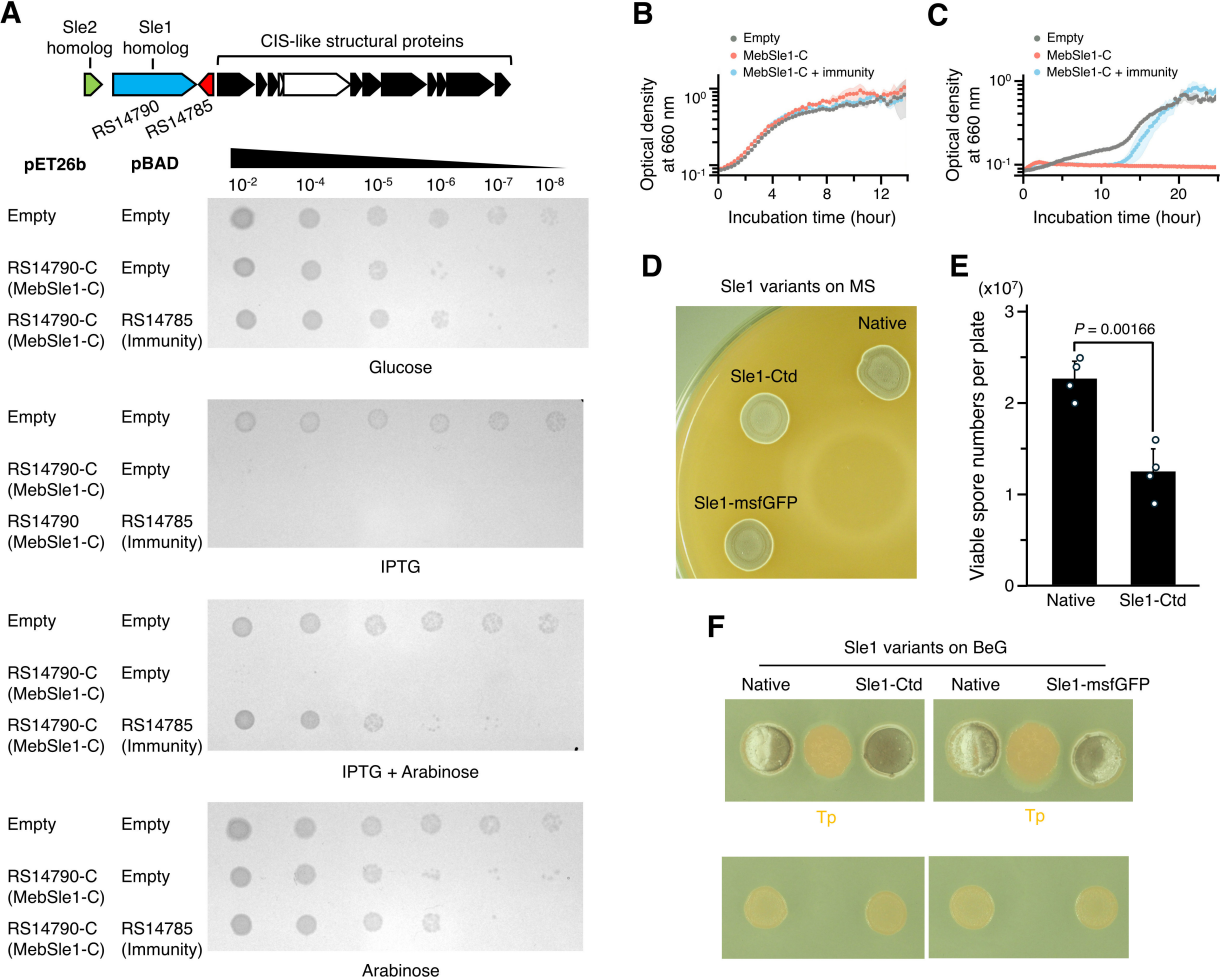
Functional diversification of Sle1-type effectors. The functional domain of the Sle1-like protein associated with the CIS-related gene cluster of *M. eburnea* was identified, and the effect of appending this domain to Sle1 was investigated. (**A**) The *E. coli* strains harboring either of the pET26b/pBAD plasmid sets were cultured, serially diluted, and spotted onto solid LB medium containing 50 μg/mL antibiotics and the additives (0.2% [wt/vol] glucose as a repressor; 0.15 mM IPTG as an inducer for pET26b; 0.2% [wt/vol] arabinose as an inducer for pBAD). (**B**) The *E. coli* strains used in panel A were cultivated in liquid LB-Lennox medium containing kanamycin and ampicillin. 0.5% (wt/vol) glucose and arabinose were added to the media as a suppressor and inducer for pET26b and pBAD, respectively. (**C**) Glucose in the cultures in panel B was replaced with 0.25 mM IPTG to induce protein expression from pET26b. (**D**) 10^4^ viable spores of each strain were spotted onto a solid MS medium and incubated for 4 days. Note that spore maturation of *S. lividans* colony involves colony color turning from white into gray. (**E**) Spores were collected from colonies grown on solid MS medium, and then the numbers of viable spores were measured. Values and error bars indicate means ± S.D. for four independent cultures. *P* value was calculated by *t*-test with Welch’s correction. (**F**) *S. lividans* strains were co-cultured with *T. pulmonis* on a solid medium without direct contact for 5 days. The white and gray regions of the colonies indicate aerial mycelia and subsequent spore maturation, respectively.

Importantly, the C-terminal regions of the identified Sle1-type proteins showed little similarity to each other, implying the functional diversification of the putative Sle1-type effectors. To examine this possibility, we cloned the C-termini of Sle1-type proteins RS14790 from *Micromonospora eburnea* DSM 44814 and RS24535 from *Amycolatopsis mediterranei* U32. Whereas RS24535 showed no detectable effect, RS14790 significantly inhibited *E. coli* growth presumably by its enzymatic activity on nucleic acids (Fig. 5A; Fig. S19 and S20). Furthermore, we also found that a hypothetical protein RS14785 encoded just downstream of RS14790 significantly alleviated the growth inhibition caused by RS14790 upon co-expression (Fig. 5A through C). Thus, these proteins would constitute an effector-immunity system associated with an SLP-like nanomachine. Notably, an Sle1-associated immunity protein was not found in the SLP gene cluster, suggesting that the RS14790/RS14785 system is functionally distinct from Sle1, although it retains the key features of Sle1-type effectors.

### Sle1 can alter the phenotypic response of *S. lividans* to neighboring bacteria by acquiring an exogenous functional module

To test whether functional diversification of Sle1-type effectors influences the phenotype of a producer bacterium, we fused the cytidine deaminase-like domain (Ctd) of RS14790 from *M. eburnea* with the C-terminus of Sle1 and introduced the resultant artificial Sle1 variant, Sle1-Ctd, into *S. lividans*. The strain possessing Sle1-Ctd formed slightly whitish colonies on the spore formation medium (MS medium) compared to the control strains possessing either the native Sle1 or the msfGFP-fused Sle1, suggesting limited spore maturation (Fig. 5D) (32, 33). Consistent with this observation, the formation of viable spores was significantly lower in the strain possessing Sle1-Ctd than in the control strain (Fig. 5E). Since these strains showed no other apparent phenotypic differences in monoculture, we tested co-culture with *T. pulmonis*, which promoted visible phenotypes associated with Sle1 (Fig. 3). In contrast to the phenotypes observed in the monoculture, the strain expressing Sle1-Ctd formed gray regions on the colony more rapidly upon approaching than the control strains, indicating faster formation of mature spores (Fig. 5F; Fig. S21). These results suggest that functional modification of Sle1 can alter the phenotypic response of *S. lividans* to neighboring bacteria.

## DISCUSSION

A series of data suggests that Sle1, encoded just upstream of the sheath protein SlpS, is a cargo loaded into SLP. Sle1 contains the coiled-coil region spanning 440 amino acids at the N-terminus, which is physically and genetically associated with DUF4157 domain-containing proteins and shows remote homology with the coiled-coil regions of phage tape measure proteins. Although the detailed loading mechanism of tape measure proteins is not fully understood, several cryo-EM studies have shown that the coiled-coil segments of tape measure proteins interacting with the spike-baseplate complex extend along the lumen and are surrounded by organized tube protein complexes (34–37). Tape measure proteins are ejected upon sheath contraction of tailed phages and are then inserted into the cellular membrane, thereby playing a role in the transfer of viral genetic material into target cells (38–41). It is noteworthy that the tape measure protein-related effector (Tme) in *Streptomyces davawensis* shares the basic action mechanism with phage tape measure proteins, suggesting an evolutionary relationship between certain CIS effectors and the phage cargo (21). The findings of our study would expand the generality of this concept to a broader range of CIS effectors, including Sle1. However, the lack of the CIS core domain DUF4157 was a significant difference distinguishing Sle1 from Tme-like effectors, implying distinct molecular mechanisms underlying their associations with SLP/CIS. Thus, Sle1 would represent a novel class of tape measure protein-like CIS effectors, providing the new definition of CIS effectors (13, 14, 20, 23).

As recently demonstrated in a tape measure protein of another phage tail-like nanomachine (37), the predicted globular C-terminal domain of Sle1, which consists of alpha-helices connected by turns/loops, may adapt its conformation to the tube lumen before ejection (Fig. 1E). Upon sheath contraction, Sle1 is ejected from the particle, and then the C-terminal domain would be relieved of the spatial constraints of the tube lumen and fold into a globular structure. Given the presence of the predicted transmembrane helices in the C-terminal domain of Sle1 (Fig. S4), Sle1 might be translocated to the cellular membrane, and SLP may facilitate this process (Fig. 1J and K). Regarding this possibility, it should be noted that the closely related CIS*^Sc^* might transiently interact with a putative membrane-associated protein which possibly induces sheath contraction and membrane insertion of an effector(s) (42). Because the putative membrane-associated protein (SLIV_17175, Fig. 1A) is also conserved in the SLP gene cluster, Sle1 may be translocated to the cellular membrane through a similar mechanism (Fig. 1A). Furthermore, the potential interactions with ribosome-containing fractions would suggest that Sle1 might act as a molecular hub linking the protein synthesis machinery and cellular membrane, thereby facilitating the expression of membrane proteins transcribed in the proximity of Sle1, although this working hypothesis would need further verification (Fig. 2F through K). The mechanistic link between Sle1 and ribosome could also be indirect, and we do not rule out the contribution of other factors (e.g., RNA or other ribonucleoprotein complexes) in the observed proteome shift. Tape measure proteins of some actinobacterial phages have long been known to contain C-terminal motifs that potentially modulate host physiology for phage reproduction, implying their alternative roles as viral signaling molecules (43–45). Thus, it is possible that the remarkable tolerance of phage tape measure proteins to structural variations might have allowed for the emergence of Sle1-type effectors with unique biological functions.

The *Streptomyces* life cycle encompasses spore germination and substrate mycelial extension along and beneath the surface of the medium, eventually forming aerial mycelia that extend into the air. The transition from vegetative growth of substrate mycelia to aerial mycelial erection often occurs in response to external nutritional deprivation (27). Therefore, this unique process has to largely rely on the reuse of cellular building blocks and the consumption of energy sources stored within the biomass of substrate mycelia at the late stage of vegetative growth (46). Aerial mycelia finally form unicellular spores that are more persistent under nutrient limitations and are crucial for increasing the overall fitness of *Streptomyces* colonies (47). Therefore, active substrate mycelial biomass would be one of the factors determining colony fitness, and its importance in *Streptomyces* ecology could be more significant in natural settings, where microorganisms compete spatially for limited nutrition. From this perspective, our findings on Sle1 may suggest its possible role in maintaining active biomass containing building blocks and energy sources that can be readily utilized for spore formation under competitive conditions. Sle1 would direct protein synthesis to the relatively small membrane-associated proteome subset while decreasing the larger subset rich in cytoplasmic proteins, possibly avoiding excessive resource consumption (Fig. 2H through K). Among the membrane proteins increased in abundance in the parental strain, those crucial for aerobic respiration would be notable since their reduction in the relative abundance may explain the decreased metabolic activity of the Δ*slpS* and Δ*sle1/2* mutants (Fig. 3; Supplemental data). Although functions of the enriched hypothetical membrane proteins remain unclear, we speculate that they might contribute to the proper localization and/or stabilization of other membrane proteins, including those essential for energy metabolism and the maintenance of cell envelope integrity. Additionally, the robust regulatory linkages between SLP production and nutritional and cell envelope stress (16, 17, 19, 48) support the idea that the significance of SLP and its effector Sle1 is relevant to the fitness of *S. lividans* under ecological stress, which could compensate for the high metabolic cost of producing this nanomachine (49).

Our results also have ecological implications of the diversification of Sle1-type effectors among actinobacterial species. We show that the fusion of Sle1 with the deaminase-like domain led to faster spore maturation in response to neighboring bacteria, indicating that Sle1-type effectors can affect producer phenotypes under competition by acquiring a new functional module at the C-terminus. Although it remains unclear how the deaminase-like domain alters the pattern of spore formation, we assume that moderate genotoxic stress caused by this domain may activate stress response pathways and render *S. lividans* more prepared to proceed with sporulation in response to microbial competition. Such mutation in Sle1-type effectors could be beneficial under certain ecological circumstances, eventually leading to the conservation of functional motifs/domains in these effectors and ultimately their diversification. The evolutionary selection of CIS effectors may also explain the variations in CIS-associated phenotypes among *Streptomyces* species (Supplemental notes). While the current study suggests the above ecological relevance of Sle1-type effectors, deeper investigation will be required to draw a conclusion.

Taken together, the present study identified the novel group of effectors associated with phage tail-like nanomachines that are highly conserved in actinobacterial species. The inferred evolutionary background of these effectors suggests that, in the end of the ancient host-virus arms race, the potential of the phage infection machinery has been unleashed and harnessed as a reconfigurable tuner for cellular functions. Our findings shed light on the previously untapped resource of CIS effectors and connect them to ecological trait modulation, expanding the applicability of the CIS repertoire as versatile biotools.

## MATERIALS AND METHODS

### Culture conditions

Strains used in this study are listed in Table S1. *M. eburnea* NBRC 101912 and *A. mediterranei* NBRC 13415 were obtained from NBRC (Chiba, Japan). *Streptomyces lividans* TK23 and *S. griseus* IFO13350 were routinely grown in Bennett’s-glucose medium comprising 0.1 g yeast extract, 0.1 g meat extract, 0.2 g N-Z amine, and 1 g glucose per 100 mL (pH 7.2). For solid media, 2% (wt/vol) agar was added. For spore formation, *Streptomyces* species were grown on mannitol-soya flour (MS) medium comprising 2 g mannitol, 2 g soya flour, and 2 g agar per 1 L. For extraction of SLP and lipid membranes, *S. lividans* spores (approximately 10^4^ viable spores) were inoculated on a sterilized cellophane membrane placed onto Bennett’s-glucose medium, and then, the colonies were scraped off the membrane for further treatment. For growth measurements, *S. lividans* was precultured in 4 mL Bennett’s-glucose medium, and then the preculture was inoculated into 100 mL YPD medium comprising 1 g yeast extract, 2 g peptone, and 2 g glucose per 100 mL. Note that YPD medium was used to obtain reproducible growth curves of this bacterium. *T. pulmonis* was grown in liquid tryptic soy broth medium for preculturing. *E. coli* strains were grown in LB-Lennox medium comprising 5 g yeast extract, 10 g tryptone, and 5 g NaCl per 1 L.

### Genetic manipulations

Primers and plasmids used in this study are listed in Table S2. NEBuilder (New England Biolabs, MA, USA) and In-Fusion (Takara Bio Inc., Shiga, Japan) were used for sequence-independent DNA cloning. KOD One (Takara Bio Inc.), Prime STAR Max (Takara Bio Inc.), and Ex Premier (Takara Bio Inc.) were used for PCR.

For markerless in-frame gene deletion, flanking regions of *SLIV_17110* or *SLIV_17110-SLIV_17115* were amplified by PCR using primer sets (Sle2-FR1_Fw/Rv; Sle2-FR2_Fw/Rv; Sle1/2-FR2_Fw_Rv) and fused with pK18mob plasmid digested with EcoRI and HindIII. The resultant plasmids (pk18mob::*sle2*-FR12 and pk18mob::*sle1/2*-FR12) were amplified in *E. coli* DH5α, and then each of the plasmids was introduced into *E. coli* S17-1. The transformed *E. coli* was grown in liquid LB-Lennox medium containing 50 μg/mL kanamycin and 50 μg/mL streptomycin, and cells at the early exponential growth phase were collected from 1 mL of the culture. The collected *E. coli* cells were resuspended with *S. lividans* spore solution (approximately 10^7^ viable spores), and the mixture was spread onto a MS medium. After incubation at 30°C for 16–20 h, kanamycin and aztreonam were added to the culture to the final concentrations of 20 μg/mL. The transconjugants that appeared after additional incubation for 2–3 days were isolated and streaked onto a fresh Bennett’s-glucose medium containing kanamycin and aztreonam. The sub-cultured colonies were grown on a solid MS medium for spore formation and the spores were spread onto solid Bennett’s-glucose medium with dilution to approximately 100 spores per plate. The deletion mutants were selected from the resultant colonies by a kanamycin sensitivity assay and colony PCR.

For integration of gene cassettes into the *S. lividans* chromosome, integration plasmids were constructed as follows. For the construction of the *Sle1/2* complemented strains, *Sle1-Sle2* and *Sle1-hibit* sequences including the native promoter were amplified using primer sets (Sle1/2_pTYM19t_Fw, Sle1/2_pTYM19t_Rv, Sle1(HiBiT)_Rv). For the construction of the HiBiT tag-fused Sle1, *Sle2* sequence was separately amplified using primer set (HiBiT-Sle2_Fw, Sle1/2_pTYM19t_Rv). Each of *Sle1-Sle2* and *Sle1-hibit*/*Sle2* fragments was fused with the pTYM19t (50) plasmid digested with KpnI and HindIII. For *Sle1-hibit/Sle2*, a hybridized linker sequence (GGGGSx2-HiBiT_Fw/Rv) was also added to the reaction. For the construction of the msfGFP-expressing strains, P*_tipA_*-RS, in which the ribosome binding site of the *tipA* promoter is replaced with a theophylline-inducible riboswitch (21), codon-optimized *msfgfp* was fused with pTYM19t as described above. Each pTYM19t-derived plasmid was amplified in *E. coli* DH5α and introduced into *E. coli* S17-1 for conjugation. Transconjugants were selected using thiostrepton. For the structural modification of Sle1, *msfgfp*, *RS14790-C*, *sle1*, and *sle2* sequences were amplified using primer sets (msfGFP_Sle1_Fw/Rv; RS14790-C_Sle1_Fw/Rv; Sle1/2_pTYM19t_Fw, Sle1(HiBiT)_Rv; Sle2_Fw, Sle1/2_pTYM19t_Rv) and fused with the linker sequence and the digested pTYM19t plasmid as described above.

For heterologous protein expression in *E. coli*, plasmids were constructed as follows.

For the expression using pET26b plasmid, insert sequences were amplified by PCR using primer sets (SlpT2_Fw/Rv; Slp4_Fw/Rv; Slp5_Fw/Rv; Sle1-N_Fw/Rv; Sle1-C_Fw/Rv; RS24535-C_Fw/Rv; RS14790-C_Fw/Rv) and then fused with the plasmid digested with NdeI and HindIII. If necessary, nucleic acid sequences encoding DYKDDDDK (FLAG) or DYKDHDGDYKDHDIDYKDDDDK (3 × FLAG) were fused with the insert sequences and the digested plasmid. For the expression using pET15b, insert sequences were amplified by PCR using primer sets (Sle1_pET15b_Fw/Rv; Sle2_pET15b_Fw/Rv) and then fused with the plasmid digested with NcoI and BamHI. For the expression using pColdII, insert sequences were amplified by PCR using primer set (Sle2_pCold_Fw/Rv) and then fused with the plasmid digested with BamHI and HindIII. For the expression using pMAL-c6T, the codon-optimized *sle1-C* sequence was amplified by PCR using a primer set (Sle1-C_pMAL_Fw/Rv) and then fused with the plasmid digested with NotI and HindIII. For the expression using pBAD/His A, *RS14785* sequence was amplified by PCR using primer set (RS14785_Fw/Rv) and then fused with pBAD/His A digested with NcoI and HindIII.

### Extraction of SLPs and ejection assay

SLPs were extracted from *S. lividans* mycelia as follows. Spore solutions (approximately 10^4^ viable spores) were spread onto solid Bennett’s-glucose medium. After incubation at 30°C for 2 days, the colonies were scraped off the plate and then lysed in a solution comprising 20 mM HEPES-NaOH (pH 7.5), 150 mM NaCl, 1% (vol/vol) Triton X-100, 5 mg/mL egg white lysozyme, and a protease inhibitor cocktail. After incubation at 37°C for 1 h, the solution was ultracentrifuged at 150,000 × *g* for 1 h. If necessary, the pellets were resuspended with 10 mM HEPES-NaOH (pH 7.5) and then ultracentrifuged again. The resultant pellets were resuspended in 100 μL of 10 mM HEPES-NaOH (pH 7.5) and 150 mM NaCl.

For an ejection assay, the *S. lividans* strains harboring pTYM19t::*sle1(HiBiT)-sle2* or pTYM19t::*sle1-sle2* were grown on solid Bennett’s-glucose medium and cultivated at 30°C for 2 days. Mycelia were then scraped off the plate and treated as described above to extract SLPs. Fifteen microliters of the isolated SLPs solution was mixed with the equal volume of 6 M urea or H_2_O, and then 10 μL of this mixture was diluted with 90 μL of Nano-Glo luminescence reaction reagent (Promega Corporation, WI, USA). After equilibration to room temperature, luminescence was measured by a plate reader.

### Microscopy

For transmission electron microscopy, samples were attached to thin carbon film-coated TEM grids (ALLIANCE Biosystems, Osaka, Japan) and washed with H_2_O. The samples were then visualized by negative staining.

### Proteomic analysis

Approximately 10^4^ viable spores of each of the *S. lividans* strains (TK23, Δ*slpS*, and Δ*sle1/2*) were spread onto solid Bennett’s-glucose medium and incubated at 30°C for 2 (mid-vegetative growth stage) or 3 (late-vegetative growth stage) days. The colonies were scraped off the plates and then disrupted by sonication in 50 mM HEPES-NaOH (pH 7.5). The lysates were centrifuged at 10,000 × *g* for 3 min, and the supernatants were used for further analyses. Three biologically independent samples were prepared for all the strains.

*E. coli* NiCo21(DE3) harboring either pET26b or pET26b::*3 × FLAG-sle1-C* was precultured in 4 mL liquid LB-Lennox medium containing 50 μg/mL kanamycin, and then 200 μL of the culture was inoculated into 100 mL of fresh liquid medium containing kanamycin. After cultivation with shaking at 37°C for 3 h, 0.2 mM isopropyl-β-D-thiogalactopyranoside (IPTG) was added to each culture. After further cultivation at 30°C for 3 h, cells were harvested and disrupted by sonication in 10 mM HEPES-NaOH (pH 7.5). The lysates were centrifuged at 5,000 × *g* for 3 min, and the supernatants were used for further analyses. Three biologically independent samples were prepared for all the strains.

Proteins were separated by SDS–PAGE and were treated by in-gel digestion with trypsin. The digested samples were purified by Zip-tips and were analyzed by advanced Nanoflor ultra-high-performance liquid chromatography (Bruker, MA, USA) on a Q Exactive Quadrupole Orbitrap mass spectrometer (Thermo Fisher) equipped with a Zaplous Column (0.2 i.d. × 50 mm; AMR, Inc., Japan, Tokyo) under the following conditions: column temperature, 35°C; mobile phase, gradient mixture of solvent A (0.1% formic acid) and solvent B (acetonitrile); flow rate, 1.5 mL/min; and gradient elution, 0 min (solvent A:solvent B = 95:5), 20 min (35:65), and 21 min (5:95). For protein identification, quantification, and comparison between two groups, database search was performed using a label-free quantification workflow in Proteome Discoverer 2.5, incorporating a Sequest HT search engine with Percolator, against the genomes of *S. lividans* and *E. coli*. Abundances of peptide spectral matches were averaged from the two technical replicates. Abundance ratios and *q*-values were calculated from the results for three biological replicates of each strain. The calculated abundance ratios of the detected proteins were automatically linked to the corresponding Gene Ontology terms for three categories (biological process, cellular component, and molecular function), and the proteins were grouped based on these values and terms.

We computed the point-biserial correlation between the binary “membrane protein” variable and abundance ratio as follows. For each protein, a binary variable indicating whether the protein is annotated as a membrane protein in the Gene Ontology cellular component annotation was defined. We then tested whether abundance ratio differed between proteins with membrane protein annotation and the other proteins using a two-sample *t*-test with Welch’s correction and calculated *P* value. Analyses were repeated within all the abundance ratio ranges (<0.5, 0.5–1.0, 1.0–2.0, ≥2.0).

### Protein expression and purification

*E. coli* NiCo21(DE3) cells harboring the expression plasmids were precultured in liquid LB-Lennox medium containing 0.2–1% (wt/vol) glucose and 50 μg/mL ampicillin and then inoculated into 100 or 200 mL of fresh medium containing the antibiotics. After incubation at 37°C with shaking at 150 rpm, protein expression was induced by the addition of IPTG at the final concentration of 0.2 mM. For His_6_-SLIV_17110 (Sle2), the cultures were further incubated at 18°C for 18 h. For His_6_-maltose-binding protein (MBP)-Sle1, the cultures were further incubated at 30°C for 3 h. The cells were harvested by centrifugation and then disrupted by sonication in 5 mL of the lysis buffer comprising Tris-HCl buffer (pH 8.0) and 10 mM imidazole. For purification, the filtered lysates were subjected to the Ni^2+^ affinity chromatography using His GraviTrap column. The eluates were concentrated and buffer-exchanged with 10 mM HEPES-NaOH (pH 7.5) using Amicon Ultra-10K. Purified His_6_-MBP-Sle1 was further treated with TEV protease at 4°C for 20 h and then purified by the Ni^2+^ affinity chromatography as described above. The removal of the His_6_-MBP tag was confirmed by SDS−PAGE, and purified tag-free Sle1 was concentrated and buffer-exchanged with 10 mM HEPES-NaOH (pH 7.5) for further analysis.

### *In vivo* and *in vitro* protein expression assays

*In vivo* protein expression of the *E. coli* strains was analyzed as follows. *E. coli* NiCo21(DE3) strains harboring pBAD::*msfgfp* and either pET26b or pET26b::*sle1-C* were precultured in 4 mL LB-Lennox medium containing 50 μg/mL kanamycin and ampicillin at 30°C overnight, and the cultures with optical density (660 nm) of 0.9 were inoculated to 100 mL of fresh LB-Lennox medium containing 50 μg/mL kanamycin, 50 μg/mL ampicillin, and 0.05% (wt/vol) arabinose. These cultures were incubated in 96-well plates with shaking at 30°C for 80 min, and then 0.1 mM IPTG was added. The cultures were further incubated, with optical density at 660 nm and GFP fluorescence measured every 20 min. To note, the detection settings for GFP fluorescence were adjusted to minimize the interference of background fluorescence through measuring the *E. coli* cultures without arabinose prior to the above measurement.

*In vivo* protein expression of the *S. lividans* strains was analyzed as follows. Viable spores (10^6^) of the *S. lividans* strains harboring a thiostrepton resistance gene and the P*_tipA_*-RS-*msfgfp* cassette were precultured in 4 mL Bennett’s-glucose medium at 30°C for 2 days. After cultivation, 3 mL of a fresh medium was added to each of the cultures, and 1 mL of the culture was poured into a 1.5 mL sterilized tube. After static incubation of the tubes at 30°C for 1 or 2 days, mycelia were concentrated by 10-fold by centrifugation at 3,000 × *g* for 5 min, followed by removal of 900 μL of the supernatant. Twenty microliters of the mycelial solution was added to 100 μL of a liquid Bennett’s-glucose medium in a 96-well plate with or without 20 μg/mL thiostrepton and 2 mM theophylline. After static preincubation at 30°C for 3 h, fluorescence was measured at the designated time points during incubation under the same condition. GFP fluorescence was calculated by subtracting the fluorescence of the culture without the inducers from that of the induced culture.

*In vitro* protein expression was analyzed as follows. The *E. coli* ribosome extract and other necessary components were derived from the NEBExpress Cell-free *E. coli* Protein Synthesis System (New England Biolabs). The reaction mixture was comprised of 30S extract, T7 RNA polymerase, RNase A inhibitor, pET26b::*msfgfp*, and purified Sle1-C. This reaction mixture was separated into aliquots and incubated at 30°C. The reaction was stopped by cooling on ice at the designated time points. Protein expression was quantified by measuring GFP fluorescence of the samples.

### Protein interaction assays

*In vitro* protein interactions were assayed as follows.

The *E. coli* NiCo21(DE3) strains harboring the pET26b::*3 × FLAG-SLIV17115-N(sle1-N*), pET26b::*3 × FLAG-SLIV17115-C(sle1-C*), pET26b::*FLAG-slpT2*, pET26b::*FLAG-slp4*, and pET26b::*FLAG-slp5* were precultured in 4 mL of LB medium containing 50 μg/mL kanamycin at 30°C overnight. Two hundred microliters of these precultures were inoculated into 4 mL of fresh LB medium containing 50 μg/mL kanamycin and incubated at 30°C for 1.5 h. IPTG (0.25 mM) was added to the medium and further incubated at 30°C for 3 h. The *E. coli* NiCo21(DE3) strain harboring pCold::*his6-SLIV_17110(sle2*) was precultured in 4 mL of LB medium containing 50 μg/mL ampicillin at 30°C overnight. 1 mL of the preculture was inoculated into 100 mL of LB medium containing 50 μg/mL ampicillin. After incubation at 30°C with shaking, the culture was cooled on ice, and 0.2 mM IPTG was added. The culture was further incubated at 18°C overnight. The cultures were centrifuged, and the collected cells were disrupted by sonication in 3 mL of 10 mM Tris-HCl and 10 mM imidazole (pH 8.0). The lysates were cleared by filtration with a 0.25 μm pore size. Then, 700 μL (FLAG-SlpT2, FLAG-Slp4, and FLAG-Slp5) or 2 mL (3 × FLAG-SLIV17115-N(sle1-N) and 3 × FLAG-SLIV17115-C(sle1-C)) of the lysates containing the FLAG-tagged proteins were mixed with 2 mL of the lysate containing His_6_-SLIV_17110(Sle2). The mixture was incubated at room temperature for 25 min and then subjected to the Ni^2+^-affinity chromatography using His Gravitrap column. The inputs and eluates were analyzed by western blot using the anti-DYKDDDDK antibody.

### Lipid membrane assays

Lipid membranes were purified as follows. *S. lividans* Δ*slpS* and *E. coli* NiCo21(DE3) harboring pET26b::*3 × FLAG-sle1-C* were grown on solid Bennett’s-glucose medium and liquid LB-Lennox medium at 30°C for 2 days and 3 h, respectively. The cells were collected and then disrupted by sonication in 10 mM HEPES-NaOH (pH 7.5). After removing debris by a brief centrifugation, each of the supernatants was ultracentrifuged at 150,000 × *g* for 60 min. Each of the pellets containing the isolated lipid membranes was resuspended in 15% (wt/vol) iodixanol and then layered on top of an iodixanol gradient (20–50%) in a tube. After ultracentrifugation at 100,000 × *g* for 3 h, the iodixanol gradient was fractionated, and lipid membrane content of each fraction was quantified by staining FM-143 dye and measuring its fluorescence. The lower density fractions containing lipid membranes were diluted with 10 mM HEPES-NaOH buffer (pH 7.5) and then ultracentrifuged at 150,000 × *g* for 60 min. The resultant pellet was resuspended in 10 mM HEPES-NaOH (pH 7.5) and used as purified lipid membranes for further analyses.

For analyzing the interaction between lipid membranes and SLP, purified lipid membranes and SLPs were mixed and incubated at room temperature for 1 h and then at 4°C overnight. To note, SLPs were extracted as described under *Extraction of SLPs and ejection assay* and washed with 10 mM HEPES-NaOH (pH 7.5) to remove remaining detergent in the extract before use for the interaction assay with lipid membranes. The mixture was subjected to the iodixanol density-gradient ultracentrifugation as described above to separate lipid membranes and SLPs that are supposed to migrate to the lower and higher density fractions, respectively. The density gradient was fractionated and subjected to western blotting analysis, FM1-43 staining, and the luminescence assay.

### Bioinformatic analyses

Amino acid sequences of SLP-related proteins were retrieved from eCIStem (20) and the NCBI database. Protein structures were modeled using AlphaFold3 (51). Domain search and homology search were performed by the NCBI Conserved Domains Database (52), the Basic Local Alignment Search Tool (BLAST) (53), and HHpred (structural model: PDB_mmCIF70_25_May) (54). Coiled-coil prediction was performed using WaggaWagga (55) and CoCoPRED (56). Transmembrane helix prediction was performed by TOPCONS (57).

### Western blotting

Rabbit antisera against SlpS, SlpT2, and Sle1 were developed using internal peptides RNDSERGVHKAPAN, CEGLSTQVEVEQRQEGGNNG, and SEANAATKRQRSSLEEAG, respectively, as antigens. Proteins were separated by SDS–PAGE using a 4–15% Mini-PROTEAN TGX Gel (Bio-Rad Laboratories, Inc., CA, USA) and then electroblotted onto a PVDF membrane. Transblotting was performed using Tris/glycine system with 10% (vol/vol) methanol or the Trans-Blot Turbo Transfer System (Bio-Rad Laboratories). After blocking with 5% (wt/vol) skim milk in Tris-buffered saline supplemented with 0.02% (vol/vol) Tween 20 (TBS-T buffer), the blots were incubated with each antibody (anti-SlpS, -SlpT2, and -Sle1 sera, diluted to 0.1%; anti-DYKDDDDK antibody [Proteintech, IL, USA], diluted to 0.01%) at room temperature for 60 min, followed by horseradish peroxidase-conjugated secondary antibody (goat anti-rabbit IgG H&L [HRP] ab6721[Abcam, Cambridge, UK], diluted to 0.01%) at room temperature for 45 min. If necessary, the primary antibodies were incubated with the blots at 4°C overnight. All the antibodies were diluted in the blocking buffer. The immunoreactive proteins were detected using ImmunoStar LD (FUJIFILM Wako Chemicals, Osaka, Japan).

### Co-immunoprecipitation assay

*E. coli* NiCo21(DE3) harboring either pET26b::*sle1-C* or pET26b::*3 × FLAG-sle1-C* was precultured in 4 mL liquid LB-Lennox medium containing 50 μg/mL kanamycin and 0.5% (wt/vol) glucose, and then the cultures were transferred to 100 mL LB-Lennox medium containing kanamycin. These induction cultures were preincubated at 30°C for 30 min, and then 0.3 mM IPTG was added to the cultures. After further cultivation for 5 h, the cells were harvested by a brief centrifugation and disrupted individually by sonication in a buffer comprising 10 mM HEPES-NaOH (pH 7.4), 150 mM NaCl, and 0.5 mM EDTA. After 0.45 μm filtration, the protein extracts were mixed with DYKDDDDK Fab-Trap Agarose Beads (Proteintech), and interacting proteins were isolated following the protocol provided by the supplier. The proteins were eluted from the beads with Laemmli sample buffer containing 10% (vol/vol) 2-mercaptoethanol by incubating at 95°C for 5 min. The eluted proteins were separated by SDS−PAGE, and CBB-visualized bands of interest were excised from the gel. The isolated proteins were digested with trypsin, and a carbamidomethyl group was added to the –SH group of cysteine residues of the peptides. The resulting peptides were analyzed by matrix-assisted laser desorption ionization-time of flight (MALDI-TOF) mass spectrometry. The obtained mass spectra derived from these peptides were analyzed by a MASCOT database search (Matrix Science Ltd., London, UK). Proteins with the scores above the significance threshold were assigned to the bands.

### Growth measurements

The *E. coli* strains were precultured in 4 mL LB-Lennox medium containing selection antibiotics and cultivated at 30°C until the cultures entered the stationary phase. They were inoculated into fresh medium and incubated at 30°C in a 96-well plate with vigorous shaking, and optical density at 660 nm and GFP fluorescence were measured using a plate reader during cultivation. For the functional assay of RS14790 and RS14785 in *E. coli*, the strains harboring each of the plasmid sets derived from pET26b or pBAD/His A were precultured containing appropriate antibiotics, and then, the preculture was inoculated into fresh medium containing the antibiotics and inducers. The cultures were incubated at 30°C with shaking. Optical density was measured using a plate reader.

Growth of *S. lividans* was measured as follows. Viable spores (10^6^) of the *S. lividans* strains were inoculated into 4 mL liquid Bennett’s-glucose medium and cultivated at 30°C for 2 days. Then, 1 mL of the preculture was inoculated into 100 mL of YPD medium. During cultivation with shaking (150 rpm) at 30°C, 1 mL of the culture was sampled at the designated time points, and the dry cell weight was measured immediately after sampling.

### Ribosome isolation

*S. lividans* TK23 was grown in 100 mL liquid Bennett’s-glucose medium, and substrate mycelia were harvested by centrifugation. The mycelia were resuspended in 500 µL of cold resuspension buffer comprising 20 mM Tris-HCl (pH 7.5), 15 mM MgCl_2_, and 1 mg/mL lysozyme. The resuspension solution was frozen and thawed three times to gently disrupt the cells. Next, 15 µL of 10% (wt/vol) sodium cholate and 10 µL of 1 mg/mL DNase I were added, and the solution was centrifuged at 5,000 × *g* for 15 min to remove debris. The supernatant was placed on top of a sucrose-gradient solution in an ultracentrifugation tube. The sucrose-gradient solution was prepared by layering 0 and 50% (wt/vol) sucrose solutions containing 10 mM Tris-HCl (pH 7.5), 50 mM NaCl, 50 mM KCl, 10 mM MgCl_2_, and 6 mM β-mercaptoethanol, and mixing the layered solutions using a Gradient Station (BioComp Instruments, Inc., New Brunswick, Canada). The sucrose-gradient solution was ultracentrifuged at 111,000 × *g* for 4 h and fractionated. UV adsorption was measured using a Triax Flow Cell (BioComp Instruments, Inc.) connected to the fractionator.

### Cell viability assay

Viable spores (10^6^) of the *S. lividans* strains were inoculated into 4 mL of Bennett’s-glucose medium and cultivated at 30°C for 2 days. After cultivation, 3 mL of a fresh medium was added to each of the cultures, and 300 μL of the culture and 200 μL of a fresh medium were mixed in a 1.5 mL sterilized tube. After static incubation of the tubes at 30°C for 1 or 2 days, 25 μL of the culture was mixed with 35 μL of fresh medium and 40 μL of a luminescence reaction reagent reconstituted from RealTime-Glo MT Cell Viability Assay (Promega) in a 96-well plate. The reagent comprised 96% (vol/vol) Bennett’s-glucose medium, 2% (vol/vol) NanoLuc solution, and 2% (vol/vol) substrate solution. The plate was incubated at 30°C without shaking, and luminescence was measured every 20 min.

### Coculture assays

*S. lividans* strains harboring a thiostrepton resistance gene were competed with other microorganisms, with or without direct contact, as follows. For the direct competition, approximately 3 × 10^4^ viable spores of *S. lividans* and competitor *Streptomyces* species were inoculated onto solid MS medium and cultivated at 30°C for 5 days. After cultivation, spores were collected from the cultures, and then the numbers of viable spores of each strain were calculated by spreading the collected spores on solid TSB medium with or without thiostrepton. For the indirect competition, approximately 10^4^ viable spores of the *S. lividans* strains were spotted onto a solid Bennett’s-glucose medium, and the equivalent viable spores of competitor *Streptomyces* species or precultured *T. pulmonis* were spotted next to the *S. lividans* spores at a distance of 1 mm. The cultures were cultivated at 30°C.

## Data Availability

The referred genome sequence of *S. lividans* TK24 is available at the GenBank database (CP009124.1). Any other datasets generated for the current study are available from the corresponding authors on request.

## SUPPLEMENTAL MATERIAL

Supplemental material jb.00532-25-s0001.pdf Supplemental figures, tables, notes, and references.

Supplemental data jb.00532-25-s0002.xlsx Raw data of proteomic analysis.

ASM does not own the copyrights to Supplemental Material that may be linked to, or accessed through, an article. The authors have granted ASM a non-exclusive, world-wide license to publish the Supplemental Material files. Please contact the corresponding author directly for reuse.

## References

[1] Hampton HG, Watson BNJ, Fineran PC. 2020. The arms race between bacteria and their phage foes. Nature 577:327–336. doi:10.1038/s41586-019-1894-8 31942051

[2] Ito S, Kageyama M, Egami F. 1970. Isolation and characterization of pyocins from several strains of Pseudomonas aeruginosa. J Gen Appl Microbiol 16:205–214. doi:10.2323/jgam.16.3_205

[3] Ge P, Scholl D, Prokhorov NS, Avaylon J, Shneider MM, Browning C, Buth SA, Plattner M, Chakraborty U, Ding K, Leiman PG, Miller JF, Zhou ZH. 2020. Action of a minimal contractile bactericidal nanomachine. Nature 580:658–662. doi:10.1038/s41586-020-2186-z 32350467 PMC7513463

[4] Russell AB, Hood RD, Bui NK, LeRoux M, Vollmer W, Mougous JD. 2011. Type VI secretion delivers bacteriolytic effectors to target cells. Nature 475:343–347. doi:10.1038/nature10244 21776080 PMC3146020

[5] Backman T, Latorre SM, Symeonidi E, Muszyński A, Bleak E, Eads L, Martinez-Koury PI, Som S, Hawks A, Gloss AD, Belnap DM, Manuel AM, Deutschbauer AM, Bergelson J, Azadi P, Burbano HA, Karasov TL. 2024. A phage tail-like bacteriocin suppresses competitors in metapopulations of pathogenic bacteria. Science 384:eado0713. doi:10.1126/science.ado0713 38870284 PMC11404688

[6] Gallegos-Monterrosa R, Coulthurst SJ. 2021. The ecological impact of a bacterial weapon: microbial interactions and the Type VI secretion system. FEMS Microbiol Rev 45:fuab033. doi:10.1093/femsre/fuab033 34156081 PMC8632748

[7] Sarris PF, Ladoukakis ED, Panopoulos NJ, Scoulica EV. 2014. A phage tail-derived element with wide distribution among both prokaryotic domains: a comparative genomic and phylogenetic study. Genome Biol Evol 6:1739–1747. doi:10.1093/gbe/evu136 25015235 PMC4122934

[8] Hurst MRH, Beard SS, Jackson TA, Jones SM. 2007. Isolation and characterization of the Serratia entomophila antifeeding prophage. FEMS Microbiol Lett 270:42–48. doi:10.1111/j.1574-6968.2007.00645.x 17263838

[9] Vlisidou I, Hapeshi A, Healey JR, Smart K, Yang G, Waterfield NR. 2019. The Photorhabdus asymbiotica virulence cassettes deliver protein effectors directly into target eukaryotic cells. eLife 8:e46259. doi:10.7554/eLife.46259 31526474 PMC6748792

[10] Shikuma NJ, Pilhofer M, Weiss GL, Hadfield MG, Jensen GJ, Newman DK. 2014. Marine tubeworm metamorphosis induced by arrays of bacterial phage tail-like structures. Science 343:529–533. doi:10.1126/science.1246794 24407482 PMC4949041

[11] Jiang F, Li N, Wang X, Cheng J, Huang Y, Yang Y, Yang J, Cai B, Wang Y-P, Jin Q, Gao N. 2019. Cryo-EM structure and assembly of an extracellular contractile injection system. Cell 177:370–383. doi:10.1016/j.cell.2019.02.020 30905475

[12] Böck D, Medeiros JM, Tsao H-F, Penz T, Weiss GL, Aistleitner K, Horn M, Pilhofer M. 2017. In situ architecture, function, and evolution of a contractile injection system. Science 357:713–717. doi:10.1126/science.aan7904 28818949 PMC6485382

[13] Kreitz J, Friedrich MJ, Guru A, Lash B, Saito M, Macrae RK, Zhang F. 2023. Programmable protein delivery with a bacterial contractile injection system. Nature 616:357–364. doi:10.1038/s41586-023-05870-7 36991127 PMC10097599

[14] Jiang F, Shen J, Cheng J, Wang X, Yang J, Li N, Gao N, Jin Q. 2022. N-terminal signal peptides facilitate the engineering of PVC complex as a potent protein delivery system. Sci Adv 8:eabm2343. doi:10.1126/sciadv.abm2343 35486720 PMC9054023

[15] Chen L, Song N, Liu B, Zhang N, Alikhan N-F, Zhou Z, Zhou Y, Zhou S, Zheng D, Chen M, Hapeshi A, Healey J, Waterfield NR, Yang J, Yang G. 2019. Genome-wide identification and characterization of a superfamily of bacterial extracellular contractile injection systems. Cell Rep 29:511–521. doi:10.1016/j.celrep.2019.08.096 31597107 PMC6899500

[16] Nagakubo T, Yamamoto T, Asamizu S, Toyofuku M, Nomura N, Onaka H. 2021. Phage tail-like nanostructures affect microbial interactions between Streptomyces and fungi. Sci Rep 11:20116. doi:10.1038/s41598-021-99490-8 34635733 PMC8505568

[17] Nagakubo T, Asamizu S, Yamamoto T, Kato M, Nishiyama T, Toyofuku M, Nomura N, Onaka H. 2023. Intracellular phage tail-like nanostructures affect susceptibility of Streptomyces lividans to osmotic stress. mSphere 8:e0011423. doi:10.1128/msphere.00114-23 37039698 PMC10286715

[18] Casu B, Sallmen JW, Schlimpert S, Pilhofer M. 2023. Cytoplasmic contractile injection systems mediate cell death in Streptomyces. Nat Microbiol 8:711–726. doi:10.1038/s41564-023-01341-x 36894633 PMC10066040

[19] Kim DW, Chater KF, Lee KJ, Hesketh A. 2005. Effects of growth phase and the developmentally significant bldA-specified tRNA on the membrane-associated proteome of Streptomyces coelicolor. Microbiology (Reading) 151:2707–2720. doi:10.1099/mic.0.28000-0 16079348

[20] Geller AM, Pollin I, Zlotkin D, Danov A, Nachmias N, Andreopoulos WB, Shemesh K, Levy A. 2021. The extracellular contractile injection system is enriched in environmental microbes and associates with numerous toxins. Nat Commun 12:3743. doi:10.1038/s41467-021-23777-7 34145238 PMC8213781

[21] Nagakubo T, Nishiyama T, Yamamoto T, Nomura N, Toyofuku M. 2024. Contractile injection systems facilitate sporogenic differentiation of Streptomyces davawensis through the action of a phage tapemeasure protein-related effector. Nat Commun 15:4442. doi:10.1038/s41467-024-48834-9 38789435 PMC11126660

[22] Sonani RR, Esteves NC, Scharf BE, Egelman EH. 2024. Cryo-EM structure of flagellotropic bacteriophage Chi. Structure 32:856–865. doi:10.1016/j.str.2024.03.011 38614087 PMC11246221

[23] Danov A, Pollin I, Moon E, Ho M, Wilson BA, Papathanos PA, Kaplan T, Levy A. 2024. Identification of novel toxins associated with the extracellular contractile injection system using machine learning. Mol Syst Biol 20:859–879. doi:10.1038/s44320-024-00053-6 39069594 PMC11297309

[24] Desfosses A, Venugopal H, Joshi T, Felix J, Jessop M, Jeong H, Hyun J, Heymann JB, Hurst MRH, Gutsche I, Mitra AK. 2019. Atomic structures of an entire contractile injection system in both the extended and contracted states. Nat Microbiol 4:1885–1894. doi:10.1038/s41564-019-0530-6 31384001 PMC6817355

[25] Leiman PG, Chipman PR, Kostyuchenko VA, Mesyanzhinov VV, Rossmann MG. 2004. Three-dimensional rearrangement of proteins in the tail of bacteriophage T4 on infection of its host. Cell 118:419–429. doi:10.1016/j.cell.2004.07.022 15315755

[26] Dixon AS, Schwinn MK, Hall MP, Zimmerman K, Otto P, Lubben TH, Butler BL, Binkowski BF, Machleidt T, Kirkland TA, Wood MG, Eggers CT, Encell LP, Wood KV. 2016. NanoLuc complementation reporter optimized for accurate measurement of protein interactions in cells. ACS Chem Biol 11:400–408. doi:10.1021/acschembio.5b00753 26569370

[27] Claessen D, de Jong W, Dijkhuizen L, Wösten HAB. 2006. Regulation of Streptomyces development: reach for the sky! Trends Microbiol 14:313–319. doi:10.1016/j.tim.2006.05.008 16759865

[28] Njenga R, Boele J, Öztürk Y, Koch HG. 2023. Coping with stress: how bacteria fine-tune protein synthesis and protein transport. J Biol Chem 299:105163. doi:10.1016/j.jbc.2023.105163 37586589 PMC10502375

[29] Onaka H, Mori Y, Igarashi Y, Furumai T. 2011. Mycolic acid-containing bacteria induce natural-product biosynthesis in Streptomyces species. Appl Environ Microbiol 77:400–406. doi:10.1128/AEM.01337-10 21097597 PMC3020563

[30] Asamizu S, Ozaki T, Teramoto K, Satoh K, Onaka H. 2015. Killing of mycolic acid-containing bacteria aborted induction of antibiotic production by Streptomyces in combined-culture. PLoS One 10:e0142372. doi:10.1371/journal.pone.0142372 26544713 PMC4636228

[31] Onaka H. 2025. Unlocking hidden bioactive compounds: from indolocarbazole and RiPP biosynthesis to the activation of cryptic secondary metabolism via microbial interactions. J Antibiot 78:395–407. doi:10.1038/s41429-025-00828-5 40379949

[32] Davis NK, Chater KF. 1990. Spore colour in Streptomyces coelicolor A3(2) involves the developmentally regulated synthesis of a compound biosynthetically related to polyketide antibiotics. Mol Microbiol 4:1679–1691. doi:10.1111/j.1365-2958.1990.tb00545.x 2077356

[33] McCormick JR, Flärdh K. 2012. Signals and regulators that govern Streptomyces development. FEMS Microbiol Rev 36:206–231. doi:10.1111/j.1574-6976.2011.00317.x 22092088 PMC3285474

[34] Yang F, Jiang Y-L, Zhang J-T, Zhu J, Du K, Yu R-C, Wei Z-L, Kong W-W, Cui N, Li W-F, Chen Y, Li Q, Zhou C-Z. 2023. Fine structure and assembly pattern of a minimal myophage Pam3. Proc Natl Acad Sci USA 120:e2213727120. doi:10.1073/pnas.2213727120 36656854 PMC9942802

[35] Iglesias SM, Hou C-FD, Reid J, Schauer E, Geier R, Soriaga A, Sim L, Gao L, Whitelegge J, Kyme P, Birx D, Lemire S, Cingolani G. 2024. Cryo-EM analysis of Pseudomonas phage Pa193 structural components. Commun Biol 7:1275. doi:10.1038/s42003-024-06985-x 39370451 PMC11456595

[36] Hodgkinson-Bean J, Ayala R, Jayawardena N, Rutter GL, Watson BNJ, Mayo-Muñoz D, Keal J, Fineran PC, Wolf M, Bostina M. 2025. Global structural survey of the flagellotropic myophage φTE infecting agricultural pathogen Pectobacterium atrosepticum. Nat Commun 16:3257. doi:10.1038/s41467-025-58514-x 40188083 PMC11972413

[37] Cai X, He Y, Yu I, Imani A, Scholl D, Miller JF, Zhou ZH. 2024. Atomic structures of a bacteriocin targeting Gram-positive bacteria. Nat Commun 15:7057. doi:10.1038/s41467-024-51038-w 39152109 PMC11329794

[38] Kizziah JL, Manning KA, Dearborn AD, Dokland T. 2020. Structure of the host cell recognition and penetration machinery of a Staphylococcus aureus bacteriophage. PLoS Pathog 16:e1008314. doi:10.1371/journal.ppat.1008314 32069326 PMC7048315

[39] Cumby N, Reimer K, Mengin-Lecreulx D, Davidson AR, Maxwell KL. 2015. The phage tail tape measure protein, an inner membrane protein and a periplasmic chaperone play connected roles in the genome injection process of E. coli phage HK97. Mol Microbiol 96:437–447. doi:10.1111/mmi.12918 25532427

[40] Hu B, Margolin W, Molineux IJ, Liu J. 2015. Structural remodeling of bacteriophage T4 and host membranes during infection initiation. Proc Natl Acad Sci USA 112:E4919–E4928. doi:10.1073/pnas.1501064112 26283379 PMC4568249

[41] Hu B, Margolin W, Molineux IJ, Liu J. 2013. The bacteriophage T7 virion undergoes extensive structural remodeling during infection. Science 339:576–579. doi:10.1126/science.1231887 23306440 PMC3873743

[42] Casu B, Sallmen JW, Haas PE, Chandra G, Afanasyev P, Xu J, Pilhofer M, Schlimpert S. 2025. Function and firing of the Streptomyces coelicolor contractile injection system requires the membrane protein CisA. eLife 14:RP104064. doi:10.7554/eLife.104064 40626860 PMC12237407

[43] Cornelis GR, Agrain C, Sorg I. 2006. Length control of extended protein structures in bacteria and bacteriophages. Curr Opin Microbiol 9:201–206. doi:10.1016/j.mib.2006.01.002 16458574

[44] Piuri M, Hatfull GF. 2006. A peptidoglycan hydrolase motif within the mycobacteriophage TM4 tape measure protein promotes efficient infection of stationary phase cells. Mol Microbiol 62:1569–1585. doi:10.1111/j.1365-2958.2006.05473.x 17083467 PMC1796659

[45] Pedulla ML, Ford ME, Houtz JM, Karthikeyan T, Wadsworth C, Lewis JA, Jacobs-Sera D, Falbo J, Gross J, Pannunzio NR, Brucker W, Kumar V, Kandasamy J, Keenan L, Bardarov S, Kriakov J, Lawrence JG, Jacobs WR Jr, Hendrix RW, Hatfull GF. 2003. Origins of highly mosaic mycobacteriophage genomes. Cell 113:171–182. doi:10.1016/s0092-8674(03)00233-2 12705866

[46] Allocati N, Masulli M, Di Ilio C, De Laurenzi V. 2015. Die for the community: an overview of programmed cell death in bacteria. Cell Death Dis 6:e1609. doi:10.1038/cddis.2014.570 25611384 PMC4669768

[47] Zhang Z, Du C, de Barsy F, Liem M, Liakopoulos A, van Wezel GP, Choi YH, Claessen D, Rozen DE. 2020. Antibiotic production in Streptomyces is organized by a division of labor through terminal genomic differentiation. Sci Adv 6:eaay5781. doi:10.1126/sciadv.aay5781 31998842 PMC6962034

[48] Tran NT, Huang X, Hong H-J, Bush MJ, Chandra G, Pinto D, Bibb MJ, Hutchings MI, Mascher T, Buttner MJ. 2019. Defining the regulon of genes controlled by σ^E^, a key regulator of the cell envelope stress response in Streptomyces coelicolor. Mol Microbiol 112:461–481. doi:10.1111/mmi.14250 30907454 PMC6767563

[49] Thomas L, Hodgson DA, Wentzel A, Nieselt K, Ellingsen TE, Moore J, Morrissey ER, Legaie R, Wohlleben W, Rodríguez-García A, Martín JF, Burroughs NJ, Wellington EMH, Smith MCM, STREAM Consortium. 2012. Metabolic switches and adaptations deduced from the proteomes of Streptomyces coelicolor wild type and phoP mutant grown in batch culture. Mol Cell Proteomics 11:M111. doi:10.1074/mcp.M111.013797 PMC3277767 22147733

[50] Onaka H, Taniguchi S-I, Ikeda H, Igarashi Y, Furumai T. 2003. pTOYAMAcos, pTYM18, and pTYM19, actinomycete-Escherichia coli integrating vectors for heterologous gene expression. J Antibiot 56:950–956. doi:10.7164/antibiotics.56.950 14763561

[51] Abramson J, Adler J, Dunger J, Evans R, Green T, Pritzel A, Ronneberger O, Willmore L, Ballard AJ, Bambrick J, et al.. 2024. Accurate structure prediction of biomolecular interactions with AlphaFold 3. Nature 630:493–500. doi:10.1038/s41586-024-07487-w 38718835 PMC11168924

[52] Lu S, Wang J, Chitsaz F, Derbyshire MK, Geer RC, Gonzales NR, Gwadz M, Hurwitz DI, Marchler GH, Song JS, Thanki N, Yamashita RA, Yang M, Zhang D, Zheng C, Lanczycki CJ, Marchler-Bauer A. 2020. CDD/SPARCLE: the conserved domain database in 2020. Nucleic Acids Res 48:D265–D268. doi:10.1093/nar/gkz991 31777944 PMC6943070

[53] Camacho C, Coulouris G, Avagyan V, Ma N, Papadopoulos J, Bealer K, Madden TL. 2009. BLAST+: architecture and applications. BMC Bioinformatics 10:421. doi:10.1186/1471-2105-10-421 20003500 PMC2803857

[54] Zimmermann L, Stephens A, Nam S-Z, Rau D, Kübler J, Lozajic M, Gabler F, Söding J, Lupas AN, Alva V. 2018. A completely reimplemented MPI bioinformatics toolkit with a new HHpred at its core. J Mol Biol 430:2237–2243. doi:10.1016/j.jmb.2017.12.007 29258817

[55] Simm D, Hatje K, Kollmar M. 2015. Waggawagga: comparative visualization of coiled-coil predictions and detection of stable single α-helices (SAH domains). Bioinformatics 31:767–769. doi:10.1093/bioinformatics/btu700 25338722

[56] Feng SH, Xia CQ, Shen HB. 2022. CoCoPRED: coiled-coil protein structural feature prediction from amino acid sequence using deep neural networks. Bioinformatics 38:720–729. doi:10.1093/bioinformatics/btab744 34718416

[57] Tsirigos KD, Peters C, Shu N, Käll L, Elofsson A. 2015. The TOPCONS web server for consensus prediction of membrane protein topology and signal peptides. Nucleic Acids Res 43:W401–W407. doi:10.1093/nar/gkv485 25969446 PMC4489233

