# Supplementary material for: A phage-derived reconfigurable effector associated with an actinobacterial contractile nanomachine tailors bacterial responses to competition
Source: J Bacteriol. 2026 May 6;208(6):e00532-25. doi: 10.1128/jb.00532-25 (PMC13277306; doi:10.1128/jb.00532-25)
Supplement: Supplemental material — Supplemental figures, tables, notes, and references. [file jb.00532-25-s0001.pdf]

Supplemental Material

A phage-derived reconfigurable effector associated with an actinobacterial contractile nanomachine tailors bacterial responses to competition

Nagakubo, T. *et al.*

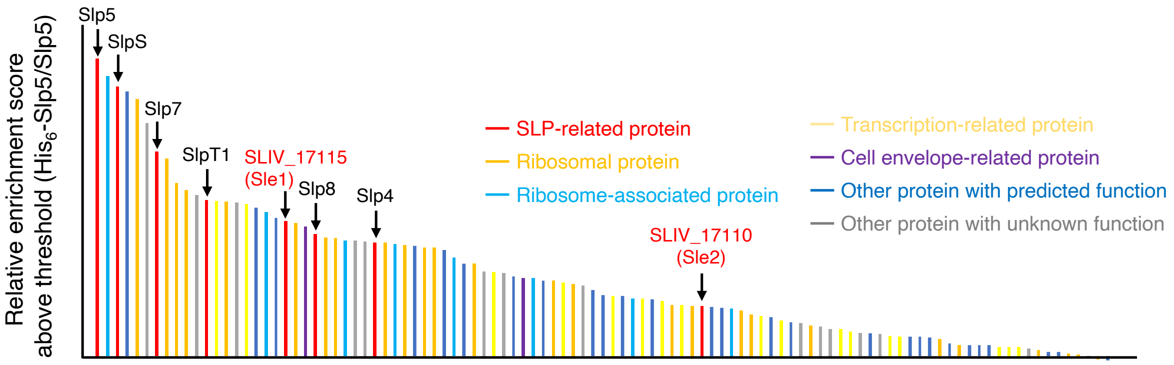

Supplemental Figure 1. Detection of SLIV_17115 (Sle1) and SLIV_17110 (Sle2)

Several proteins with unknown functions were detected in the isolated SLP fraction. This figure was created using a previously reported dataset (1). In this analysis, SLP with a His_6-tagged spike protein was isolated by Ni^{2+}-affinity chromatography, and the isolated fraction was subjected to quantitative proteomic analysis (1). The threshold for the relative enrichment score was set to >1.

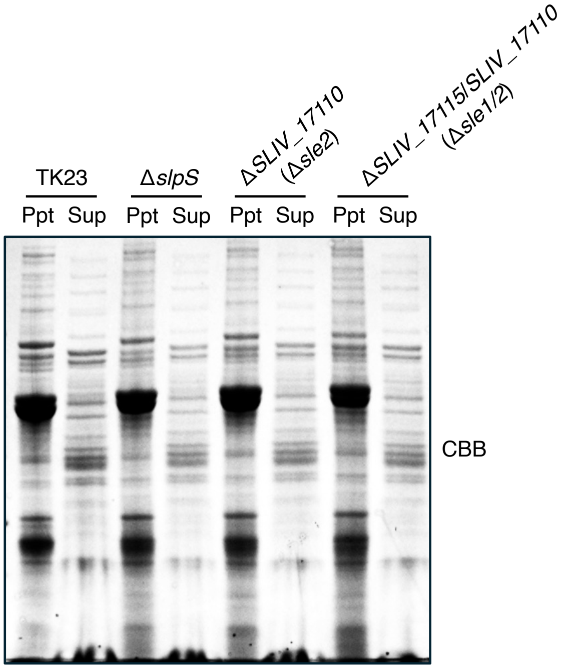

Supplemental Figure 2. CBB-stained control gel for the western blot analysis of SLIV_17115 and SlpT2

Substrate mycelia of *S. lividans* grown on a solid medium were scraped off the plate and separated into SLP fractions (ppt; ultracentrifugation pellet) and detergent-soluble fractions (sup; ultracentrifugation supernatant). These fractions were further separated by SDS–PAGE and subjected to CBB staining.

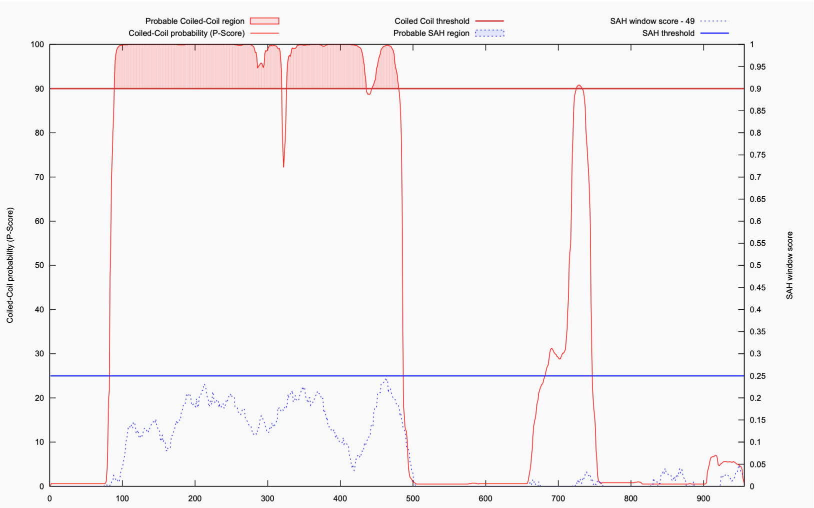

marcoil

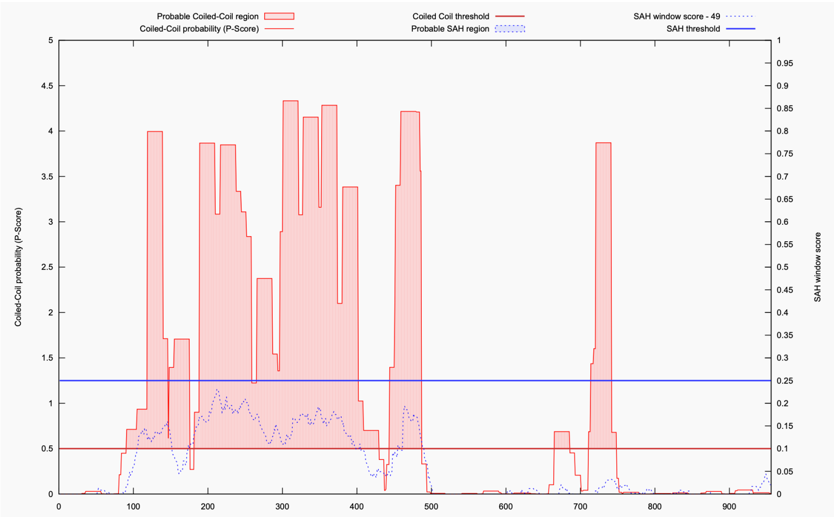

ncoils

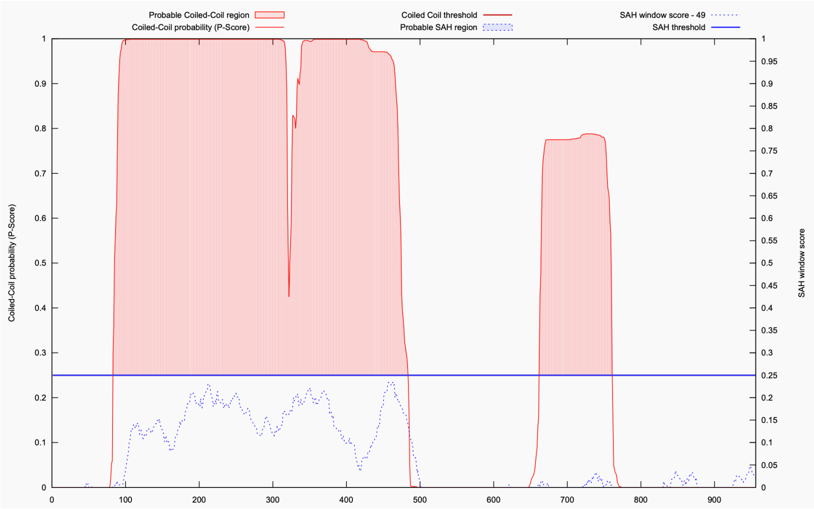

multicoil2

Supplemental Figure 3. N-terminal coiled-coil region of SLIV_17115 (Sle1)

Coiled-coil segments were bioinformatically detected in SLIV_17115 (Sle1). The figure shows the results of a comparative analysis using Waggawagga with three different prediction tools. SAH, stable single α-helix.

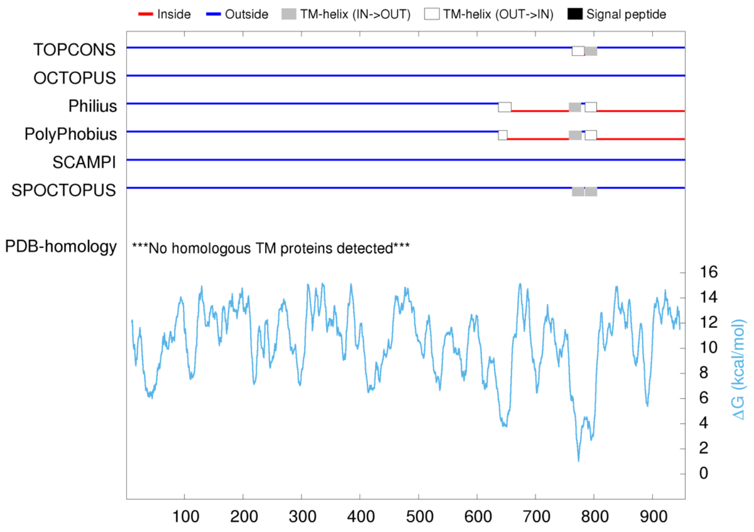

Supplemental Figure 4. Hydrophobic regions of SLIV_17115 (Sle1)

Putative transmembrane regions of Sle1 were identified by TOPCONS.

8VJH_Q Tape measure; Flagellotropic bacteriophage, Siphophage, Tail-tip, VIRUS;{Chivirus chi}

Probability: 99.67%, E-value: 9.8e-10, Score: 146.76, Aligned cols: 679, Identities: 10%, Similarity: 0.06, Template Neff: 10.[illegible]

```
Q ss_pred                HHHHHHHHHHHHHHHHHHHHHHHHHHHHHHHHHHHHHHHHHHHHHHHHHHHHHHHHHHHHHHHHHHHHHHHHHHHHHHHH
Q Q_8197009   116        TGTAIEARKKESDARDKAEALEREVGDAKRGERSSLEKARESTSEANAATKRQRSSLEEAGRHGETEKEKGAAAEEARKQ
Q Consensus   116        ~eE~eel~kql~~lq~el~qLeeekeeLeRekeElEeELe~qrkElkeA~qqRkeAeeEL~e~ee~~~el~~~kkkae~E
                         ..+++-....+..++.+++.++.++..++.++.++..+++.+..++.+...++..++.++..+......+ ..+.+++.+
T Consensus   4          ~~~~~l~i~~~~~~~~~~~~l~~~l~~l~~~~~~~~~~~~~~~~~~l~~l~~~~~~~~l~~~l~~l~~~~~-~~l~~~~~~
T 8VJH_Q      4          SKDVELRIRARDYSQKPLKAVTSAIEQMAKAQDEQRKAAERGEISTRDLEAAYKKLESAGSQLLKLNSLI-EVFKRQNQA
T ss_pred                ccceeeehhhhHhcHHHHHHHHHHHHHHHHHHHHHHHHHHHHHHHHHHHHHHHHHHHHHHHHHHHHHHHH-HHHHHHHHH


Q ss_pred                HHHHHHHHHHHHHHHHHHHHHHHHHHHHHHHHHHHHHHHHHHHHHHHHHHHHHHHHHHHHHHHHHHHHHHHHHHHHHHHH
Q Q_8197009   196        ADGFKKEAEAAEQRRKDAADLVAQLSATIEQARKSQAEAEARVRRFSRRAEEAAAQLKAADETARTAGDVEDKAQEQAAE
Q Consensus   196        ~qkLkke~eEaE~eLekaEeE~e~lrkqIrelekerke~EeeIeKL~KEkk~aee~rkkaeeElq~~edk~~ea~KqkaK
                         +..+..+++++..+++.+..++..++.++..++.++..++.++..+..++...+..+..+..++.......++.+.+..
T Consensus   83         l~~~~~~l~~~~~~~~~l~~~~~~~~~~~~~~l~~~l~~~~~~~~~l~~~~~~l~~~l~~~~~~l~~~~~~~~~~le~~~~
T 8VJH_Q      83         MTEATAKTEGLRQKQADLQKTYDSTEKVTQKQERALARVTRQVEAATRAEANQAERVNRATRDLERYGIETSKVGAAQAG
T ss_pred                HHHHHHHHHHHHHHHHHHHHHHHHHHHHHHHHHHHHHHHHHHHHHHHHHHHHHHHHHHHHHHHHHHHhHHHHHHHHHHHH


Q ss_pred                HHHHHHHHHHHHHHHHHHHHHHHHHHHHHHHHHHHHHHHHHHHH---HHHHHHHHHHHHHHHHHHHHHHHHHHHHHHHHH


Q Q_8197009   276        AEVEAESEEQFAAAMRKRTVELATALKEVKAAKEAAKRRNEEAE---GAGASQKEAEEGQEEAARLRDEANESARESAEL
Q Consensus   276        LE~ELEre~e~eeleklKkeraEAeKekrkLE~ELk~~qeelE---EaerqKkeaEeelerlekEle~L~eele~lqsE
                         ++.++   .++..+++.+..++....+.. .++.++..++..++   ........+...+..+..++..+..+++.+..+
T Consensus   163        l~~~l---~~l~~~l~~~~~~~~~~~~~~-~~~~~l~~l~~~~~~~~~~~~~~~~~~~~~~~~~~~~~~~~~l~~~l~~l~~~
T 8VJH_Q      163        IVTSV---AQVNRVLERQDEIISTSAAAA-AQAKVIRGLQQQADQAMATARGYQTLGRVVQQATGQLGPLGTQIQTIVSP
T ss_pred                HHHHH---HHHHHHHHHHHHHHHHHHHHH-HHHHHHHHHHHHHHHHHHHHHHHHHHHHHHHHHHHhchhHHHHHHHHhcH


Q ss_pred                HHHHHHHHHHHHHHHHHHHHHHHHHHHHHHHHHHHHHHHHHHHHHHHH------HHHHHHHHHHHHHHHHHHHHHHHHHH
Q Q_8197009   353        AKKLDREAKAATEARETAEQAAAGHGRTAKDAQDKAEKAQAEAAGYAK------TAGEKQTEHDAALAALTRLGREAAGA
Q Consensus   353        ~ekLqkeikElq~rreELEeELe~erqar~KAekqr~elqrELeel~~------~lEE~~~q~~~A~~e~~r~~~~a~~a
                         +..+..++..++.+++.+..++..++.....+.....++.++.++..      .+.+...++...+.++..+..+....
T Consensus   239        ~~~~~~~l~~l~~~~~~l~~~~~~l~~~~~~~~~~~l~~l~~~l~~l~~~~~~~~~~~~~~~~~l~~~~~~~~~~~~e~~~l
T 8VJH_Q      239        AEAARRTLSGLENQVHGVTAELARNSKEVENVAQKVRMLNEANKTVSALAQQIDLYRQQVATLRNARTEYQQARQDVIKL
T ss_pred                HHHHHHHHHHHHHHHHHHHHHHHHhHHHHHHHHHHHHHHHHHHHHHHHHHHHHHHHHHHHHHHHHHHHHHHHHHHHHHHH


Q ss_pred                HHHHHHHHHHHHHhccHHHHHHHHHHHHHHHHHHHHHHHHHHHHHHHHHHHHHHHHHHhcchhhhcccCCcccccchhcc
Q Q_8197009   427        RQRAAAAAQGQLRHGTAEDTARTELAAAKETEKKAAEKKAAEEKAAKEKAAKEKAAKEKEPAGAEKTDGSSRNGVARAAD
Q Consensus   427        ~~~~~~a~~~~~~~~~~~~~~~~a~~~~~~~a~~~~~~~~~~~~~~~~~~~~~~~~~~~~~~~~~~~~~~~~~~~~~~~~
                         +...............-......+...+.......   ..-......-.........-  ...-..-...-........
T Consensus   319        ~~~l~~~~~~~~~l~~~l~~~~~~~~~~~~~~~~l~---~~~~~l~~~l~~~~~~~~~l--~~~~~~l~~~~~~~~~~~~
T 8VJH_Q      319        AQQMRTATTDTGALGIQMQAAQQRLSAAATAMRNTA---TAARSTQAALRAAQVDTRNL--SDAEARLISTSQQSAAALN
T ss_pred                HHHHHhccCchHHHHHHHHHHHHHHHHHHHHHHHHH---HHHHHHHHHHHHhHhhhhcH--HHHHHHHHHHHHHHHHHHH


Q ss_pred                chhHHHHHHHhhhccccCCCCccccccCCCCCCchhhhhhhcCCccccccccccccCCChhHHHHHHHHHHHHHHHHHHH
Q Q_8197009   507        GVGHGAKKASAKTKPWADVPDTVVLRGSAPPGATVQSASMKHGDHGTAHDTVIQQSVENPINLVSDVTGTVNDIAAIKEA
Q Consensus   507        ~~~~~~~~~~~~~kk~~d~~Dt~~~~~~~~P~~ag~~~~a~~~~d~~~~~~~aa~g~~~~~~~n~v~~~~g~~~~~~~~~~a
                         .+...+..+-....+.-+..+-+...+      .........-......-+++.....+..+.++.++..+.........
T Consensus   394        ~l~~~~~~l~~~~~~~~~~~~~~~~~~~~~------~~~~~~~~~l~~~~~~~~~~~a~~~~~~~~~~~~~~~~~~~~~~~~~~~
T 8VJH_Q      394        TLSTATNRNSQAARDGSKAWSLFRDEG------RTTLSFLQRIRGEVLALTTTYVGFQGAISLAGGAIDAYKNRQQAMVK
T ss_pred                HHHHHHHHHHHHHHHHHHHHHHhhhcc------hhhHHHHHHHHHHHHHHHHHHHHHHHHHHHHHHHHHHHHHHHHHHHH


Q ss_pred                Hh-hcCCCChhhhcccccCCCchHHHHHHHHHHHHHHHHHHHHHHHHHhcHHHHHHHHHHhHHHHHH--------HHHHH
Q Q_8197009   587        AG-KRNESGPASHAHRKNYVGKPLGLASSSQMAANDVLKIANTGTKAAGDLAGTAALADAGGALTIS--------FSAIH
Q Consensus   587        ~k-~~~~~~G~~~H~a~k~~~~~k~~~a~~~~~~~~~~~~~~~~~a~~a~~~~~~~~~~~~~~~a~~~a~G~~s~~--------~~~~~
                         .. ....+.......-+....-......+...+......++..+.........+.......+.+...        .+++.
T Consensus   468        ~~~~~~~~~~~~~~~~~~~~~~a~~~g~s~~~~~~~~~~~~~~~a~~~g~~~~~~~~~~~~~~~~~~~~~g~~~~~~~~~~~
T 8VJH_Q      468        IANVVGNSQAAINKEWEYMVGLANTLGIDITTLSQSYTKFAVSAKAVGLSLQDSKFIFESVAKAGRVFHLSQDDMEGVFR
T ss_pred                HHhHhcCCHHHHHHHHHHHHHHHHHHCCCHHHHHHHHHHHHHHHHHcCCCHHHHHHHHHHHHHHHHHcCCCHHHHHHHHH


Q ss_pred                HHHHHH------------HH-hhHHHHHHHHHhccCchHHHHHHHHH-------HHHHHHHHHHHHHHHHHHccCCChhH
Q Q_8197009   658        VGRDSR------------VI-KNTHAQRKKLKDHFNDVAAKRDRKLQ-------DVLNDLGRAGVGLAERVSGLGDSEDG
Q Consensus   658        ~~R~~~~------------~~-~~a~~k~~~~Lk~~~~~~~~~~~~~~~~~~L~-------~~~~~~~~~~a~~~~~~~~~~~~~~~~~~~~~~~
                         ++-...             .+ .....-...+.+.+.........+.       .+...+...................
T Consensus   548        ~l~q~~~~g~~~~~~l~~~l~~~~~~~~~~~a~~~g~~~~~l~~~~~~G~~~~~~~~~~~~~~~~~~~~~~~~~~~~~t~~~
T 8VJH_Q      548        ALEQMLSKGQVYAEELRGQLGERLPAAFALFAKGMDMTTAELMKAMENGEVTGEAVINFAREQAKAIDAQLATAQKGVDA
T ss_pred                HHHHHHhcCCCCHHHHHHhHHhHHHHHHHHHHHHcCCCHHHHHHHHHcCCCcHHHHHHHHHHHHHHHHHHHHHHHHhHHH


Q ss_pred                -----HHHHHHHHHHHHH--HHHHHHHHHHHHHHHHHHHhHHHHHHHHHHHHHHHHHHHHHHHHHHHH-----HHHhhcC
Q Q_8197009   718        -----LKAVEDQQARVEN--LRTEVREHMAAAREYVVHKKKWKLGHRFANLTGNLARIAAGAVAIAAA-----TGAVTGG
Q Consensus   718        -----~~a~~~~r~~~~~~--~r~e~~~~L~~~~~~YA~~Kq~~k~~k~~~~~~~G~~~~~~a~G~v~iaaa-----ag~as~p
                              ..++......+..  ........+..+.++.........+++.+....+........++-....     .++++..
T Consensus   628        ~~~~l~~a~~~~~~~~~~~~g~~~~l~~~~~~~~l~~~l~~~~~~~~~~~~~l~~~~~~~l~~~~~~~~~~~~~~~~l~~~~~~~~~~~~~~~
T 8VJH_Q      628        MEARARNAMNAFQLALADAGFIEAYVQMLNKITDFLNSEDGRAAAVKLGEAFGMLADAVTWCIENVDTLITALSILAGLK
T ss_pred                HHHHHHHHHHHHHHHHHhcCHHHHHHHHHHHHHHHhcChhhHHHHHHHHHHHHHHHHHHHHHHHHHHHHHHHHHHHHHHH


Q ss_pred                hHHHHHHHHHHHHHHHHHHHHHHHHH
Q Q_8197009   786        VAPGVAGGLTAGFLGGLALKKSVKKA   811 (1044)
Q Consensus   786        vgw~lag~~~A~~~~g~a~yK~~r~~   811 (1044)
                         +..++++ ++.++...+.+++++...
T Consensus   708        ~~~~~~~-l~~~i~~~~~~~~~~~l~~~   732 (1431)
T 8VJH_Q      708        VVQFIGG-MISGLKNLLPLFNTLSKI   732 (1431)
T ss_pred                HHHHHHh-HHHHHHHHHHHHHHHHhh
```

Supplementary Figure 5. Remote homology between SLIV_17115 (Sle1) and a phage tapemeasure protein

Homology search for SLIV_17115 (Sle1) was performed by HHpred and a representative result is shown. In this figure, total amino acid sequence of Sle1 (query, Q; 1-1044) was aligned with that of tapemeasure protein of Chivirus chi (template, T; PDB accession number 8VJH_Q; 1-1431). Sequences ss_pred denote the PSI-PRED secondary structure prediction (H, helix; C, coil). Upper and lower case amino acids in the consensus sequences indicate high and moderate conservation, respectively. Symbols indicate the quality of the column-column match: |, very good; +, good; ·, neutral; -, bad.

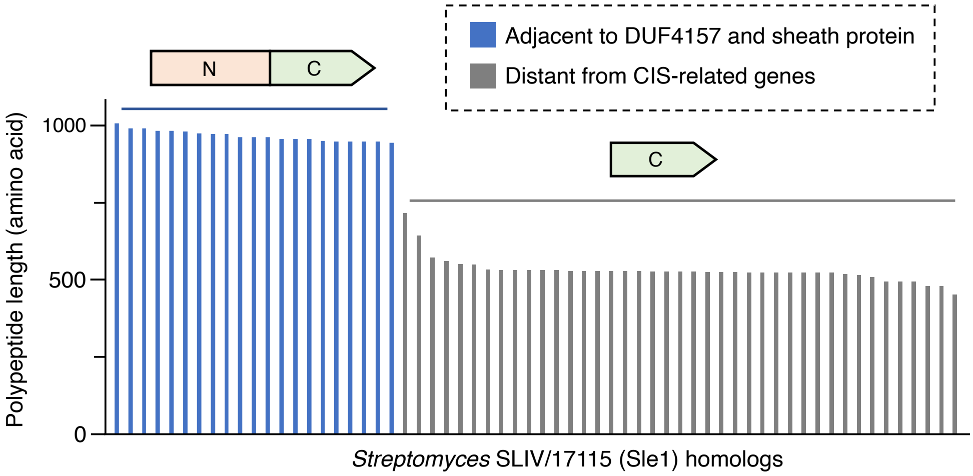

Supplemental Figure 6. The N-terminal coiled-coil region of SLIV_17115 (Sle1) is genetically associated with CIS-related genes

Close homologs of SLIV_17115 (Sle1) in the genus *Streptomyces* were identified using a BLAST search. Of these homologs, those with available RefSeq genome sequence data were extracted and arranged in order of polypeptide length. The adjacency to DUF4157 domain-containing proteins and sheath proteins was defined as the distance between the SLIV_17115 (Sle1) homologs and them within two upstream or downstream genes in the genomes.

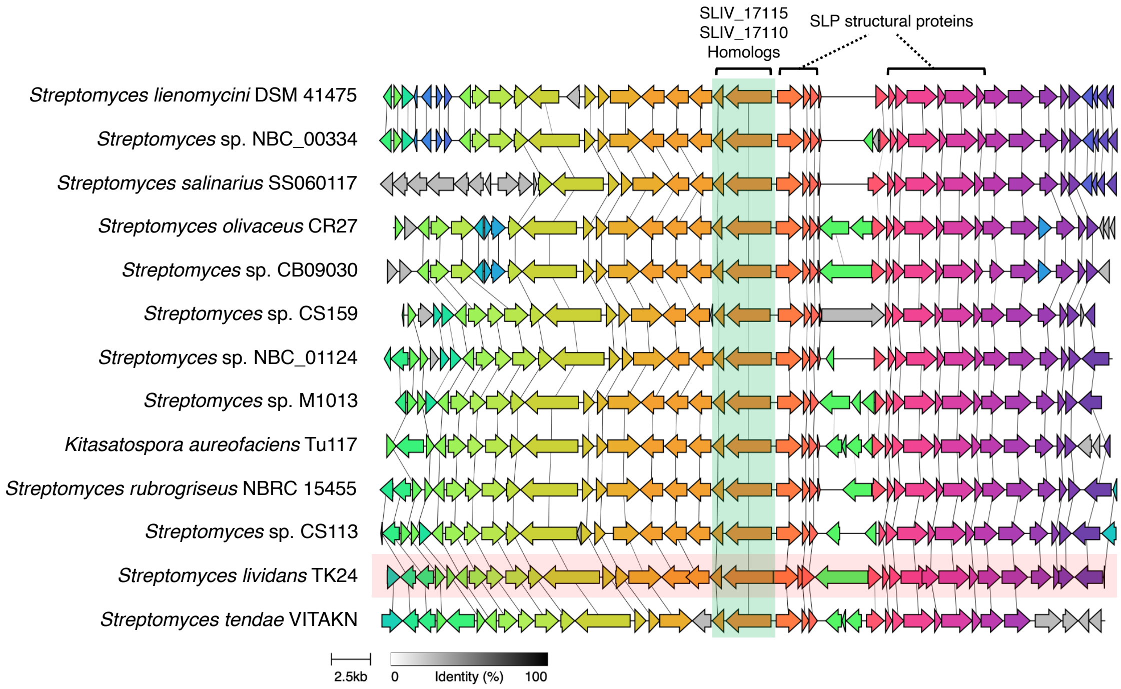

Supplemental Figure 7. Conservation of SLIV_17115 (Sle1) and SLIV_17110 (Sle2) in SLP-related gene clusters.

Close homologs of SLIV_17115 (Sle1)/SLIV_17110 (Sle2) were identified by a BALST search, and their neighbouring genes were aligned using clinker (2). Genes with relatively high similarity among the analysed genes were grouped and displayed in the same colour.

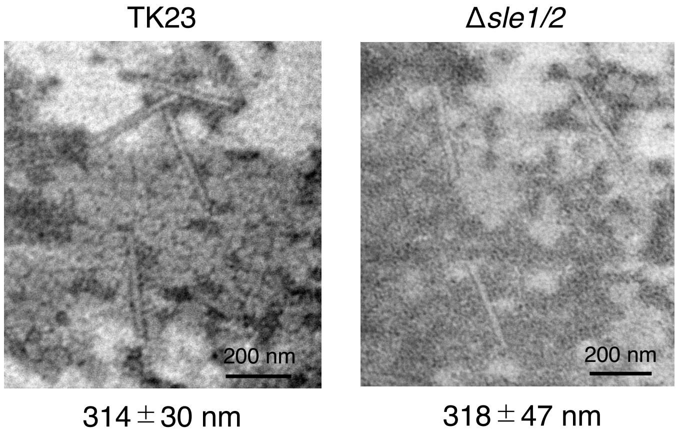

Supplemental Figure 8. Deletion of *sle1* and *sle2* does not affect SLP assembly
SLPs were extracted from substrate mycelia of the TK23 (parental) strain and the Δ*sle1/2* mutant and were observed using transmission electron microscopy. The samples were visualised by negative staining. Scale bars, 200 nm. Mean length of isolated SLPs (TK23, 80 particles; Δ*sle1/2*, 112 particles) are indicated at the bottom of each image.

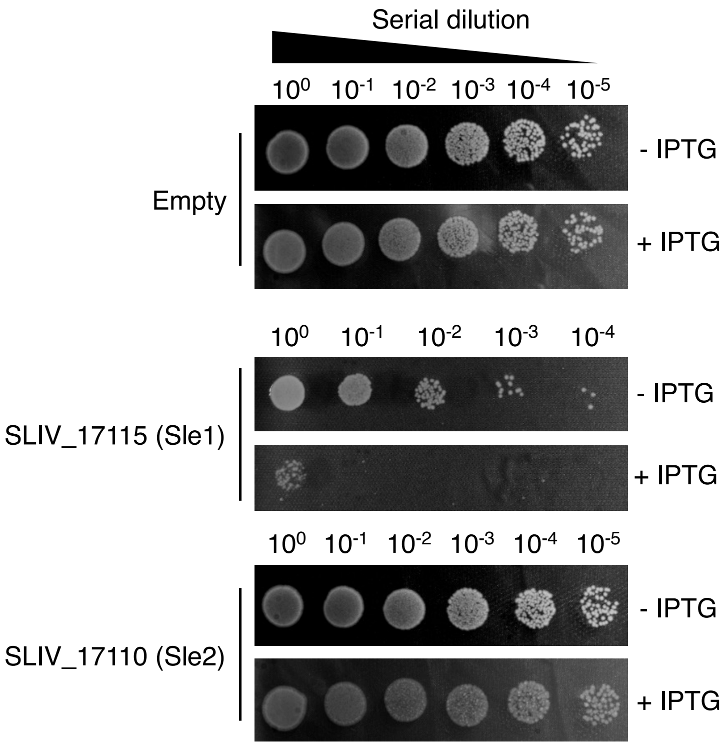

Supplemental Figure 9. Inhibition of colony formation by Sle1 expression.
E. coli BL21(DE3) habouring pET15b, pET15b::*sle1*, or pET15b::*sle2* was grown in a liquid LB medium and then the serially diluted cultures were spotted onto solid LB media with or without 1 mM IPTG. Ampicilin (50 μg/mL) was used for selection.

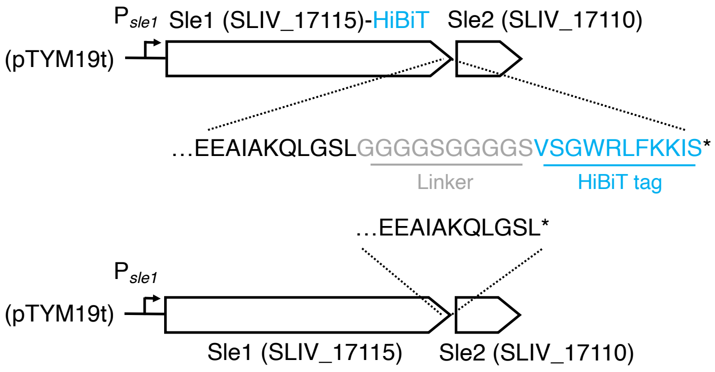

Supplemental Figure 10. Experimental design for analysing Sle1 ejection.

Sle1 was tagged at its C-terminus with a linker and HiBiT tag as shown in the figure. Sle1 and Sle2 were expressed on an integrative pTYM19t plasmid under the regulation of their native promoter. pTYM19t was integrated into the *attC* site of the *S. lividans* chromosome.

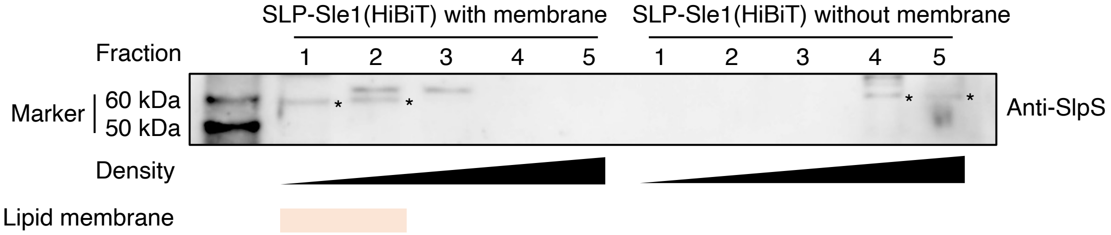

Supplemental Figure 11. Physical interaction between SLP and lipid membrane fractions.

Purified lipid membranes and isolated SLPs were mixed and subjected to density-gradient ultracentrifugation using iodixanol. The fraction numbers correspond to those in Fig. 1I and J. Lipid membrane fractions were determined by FM1-43 staining. SlpS, a sheath protein of SLP, was detected using anti-SlpS serum and the corresponding bands were determined based on its estimated molecular wight as previously reported (1).

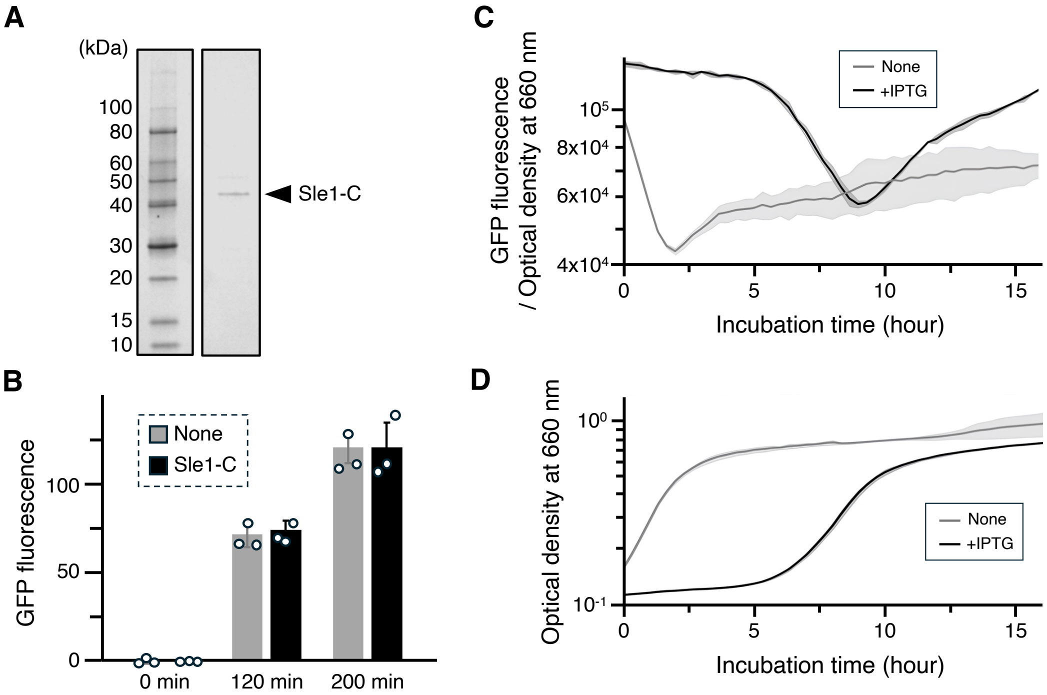

Supplemental Figure 12. Sle1-C does not negatively affect protein synthesis in *E. coli*
The protein synthesis activity of *E. coli* was measured *in vitro* and *in vivo*. (A) Tag-free Sle1-C was purified by maltose-binding protein fusion and subsequent site-specific cleavage. Details of the purification procedures are described in the Methods section. (B) *In vitro* protein synthesis activity of the *E. coli* cell lysate was measured using msfGFP as an indicator. 70 nM Sle1-C was added to the reaction mixture under the condition denoted as "Sle1-C". (C and D) *In vivo* protein synthesis activity of *E. coli* cells was measured using a dual expression system in which the expression of Sle1-C and msfGFP was induced by 0.1 mM IPTG and 0.05% (w/v) arabinose, respectively. Black and gray lines indicate the conditions with and without IPTG addition to the medium, respectively. Values are the means for biological triplicates. The area indicates the standard deviation. Optical density and fluorescence were monitored using a plate reader.

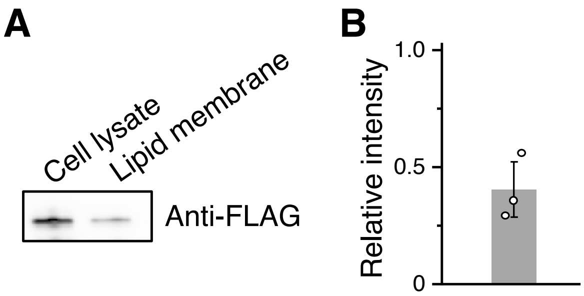

Supplemental Figure 13. Sle1-C is partially associated with the lipid membranes of *E. coli* cells.

(A) Lipid membranes were purified from *E. coli* cells expressing 3×FLAG-Sle1-C by sonication and subsequent density-gradient ultracentrifugation. Purified lipid membranes were analysed by western blotting using an anti-DYKDDDDK (FLAG) antibody. A representative blot is shown in this figure. Each sample was diluted to an appropriate volume to adjust to an equal amount of cells from which the sample was prepared and then loaded into the lane. (B) The signal intensity of 3×FLAG-Sle1-C in the purified lipid membranes relative to that in the total cell lysate was calculated from the blot images. Mean ± S.D. for three technical replicates is shown.

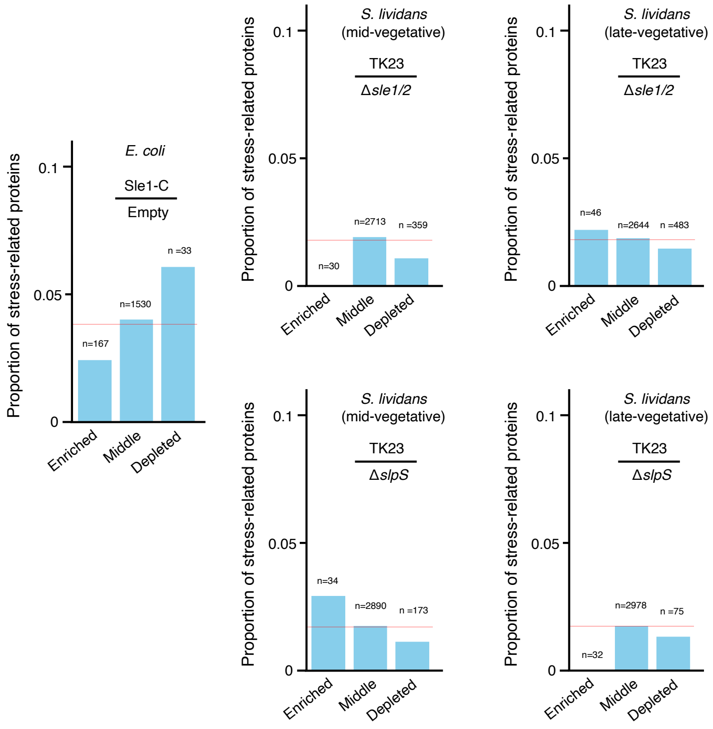

Supplemental Figure 14. Sle1 does not consistently affect the expression of stress-related proteins

The proportions of the predicted stress-related proteins detected by the proteome analysis in Figure 2 were calculated for the following three categories: enriched, abundance ratio ≥ 2; middle, -2 < abundance ratio < 2; depleted, abundance ratio ≤ -2. Stress-related proteins were extracted from the proteomic data based on the gene ontology term "stress response" in the Biological Process category. The red lines indicate the mean proportions of the predicted stress-related proteins among the total proteins commonly detected in the two strains.

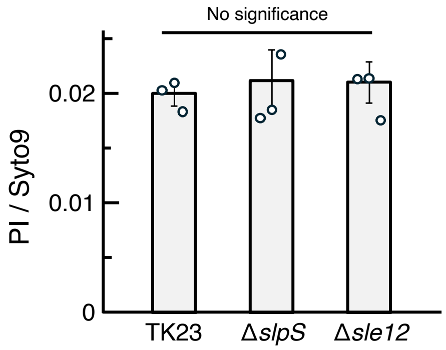

Supplemental Figure 15. Cellular membrane permeability assay for the *S. lividans* strains.

Substrate mycelia of the *S. lividans* strains were stained with Syto9 and propidium iodide (PI), which are membrane permeable and non-permeable nucleic acid-specific dyes, respectively. Mycelia were grown as Fig. 3C and the medium was replaced with the buffer containing 10 mM HEPES-NaOH (pH7.4), 1 μM Syto9, and 10 μM PI. Fluorescence of Syto9 and PI was measured using a plate reader at Ex. 470 nm/Em. 510 nm and Ex. 530 nm/Em. 620 nm, respectively. Statistical analysis using one-way ANOVA revealed no significant difference among the strains (P = 0.823).

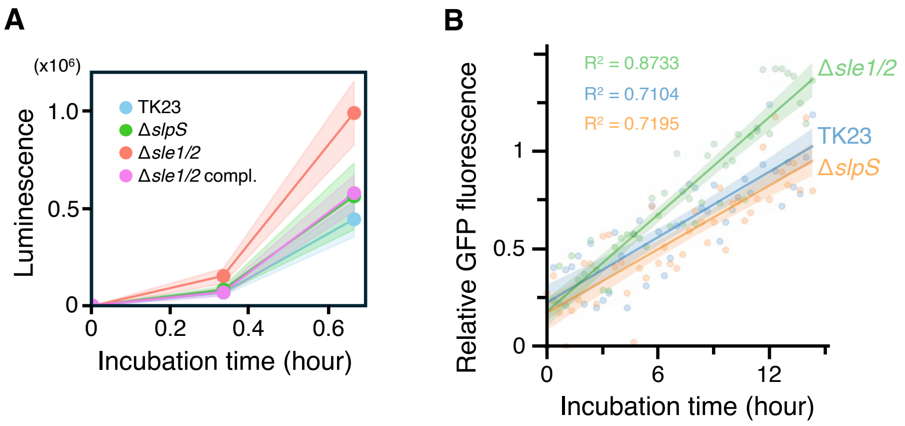

Supplemental Figure 16. The Δ*sle1/2* mutant showed relatively higher metabolic activity during the early vegetative growth stage.

(A) Cellular reducing activity of each *S. lividans* strain was measured using a cell-permeable luminescence substrate and luciferase, as described in the Methods section. Values and coloured areas indicate means ± S.D. for three independent cultures. (B) Protein synthesis capacity of *S. lividans* strains expressing msfGFP was measured. In these strains, msfGFP expression was regulated by an engineered *tipA* promoter (P_{tipA}RS), in which the transcription and translation of the downstream open reading frames were dependent on exogenously added thiostrepton and theophylline, respectively. Relative GFP fluorescence was calculated by subtracting the fluorescence of mycelia under non-inducing conditions from that under inducing conditions. The *S. lividans* strains in this panel are shown as the genetic background of the msfGFP-expressing strains. Scatter plots, lines, and coloured areas indicate the mean values for three independent samples, regression lines, and 95% confidence intervals, respectively.

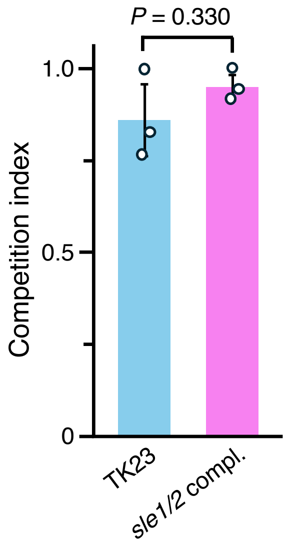

Supplemental Figure 17. Competition assay for the *sle1/2*-complemented strain.
S. lividans TK23 and Δ*sle1/2* harbouring pTYM19t and pTYM19t::*sle1/2*, respectively, were individually cocultured with *Streptomyces griseus* and then the viable spore numbers were measured using thiostrepton for selection of *S. lividans* strains. The Δ*sle1/2* harbouring pTYM19t::*sle1/2* is shown as "*sle1/2* compl." in the figure. The competition index was calculated as the viable spore number of *S. lividans* relative to that of *S. griseus*. pTYM19t was integrated into the *attC* site of the *S. lividans* chromosome.

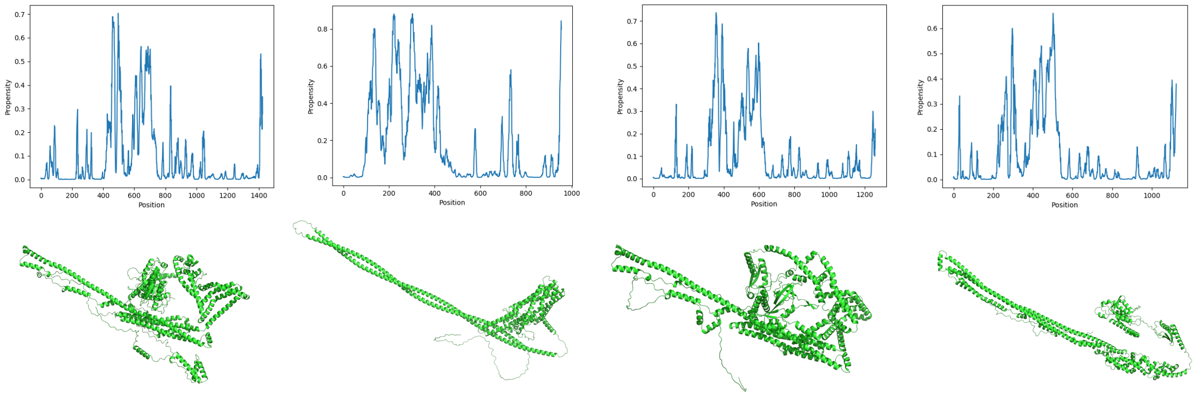

Arthrobacter sp. QXT-31 (*Staphylococcus* phage 80α TMP, 388-1108)

Streptomyces lividans TK23 (*Escherichia* Phage P2 TMP, 132-313)

Nakamurella multipartita Y-104 (*Mycobacterium* phage D29 TMP, 335-917)

Micromonospora eburnea DSM 44814 (*Clostridium* phage phiCD119 TMP, 193-782)

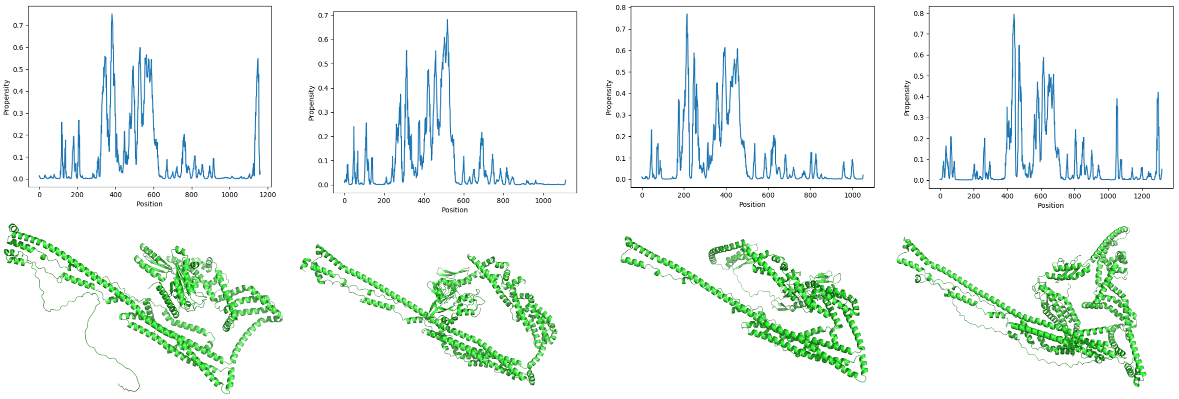

Saccharothrix sp. NRRL B-16314 (*Clostridium* phage phiCD119 TMP, 343-869)

Amycolatopsis miditerranei U32 (*Clostridium* phage phiCD119 TMP, 210-802)

Rhodococcus aetherivorans IcdP1 (*Pneumococcus* phage Dp-1 TMP, 139-734)

Mycobacterium sp. JLS (*Staphylococcus* phage 80α TMP, 360-1031)

Supplemental Figure 18. Sle1-like proteins conserved in the class actinobacteria

This figure shows the structural characteristics of Sle1-like proteins from members of the class Actinobacteria. The charts inside the squares indicate the probable coiled-coil regions of each Sle1-like protein predicted by CoCoPRED. Regions with higher "Propensity" are more likely to be coiled-coil. Ribbon diagrams are AlphaFold3-predicted structures of the Sle1-like proteins. Representative phage tapemeasure proteins with which each Sle1-like protein has remote homology and the corresponding regions are given in parentheses. Homology searches were performed using HHpred.

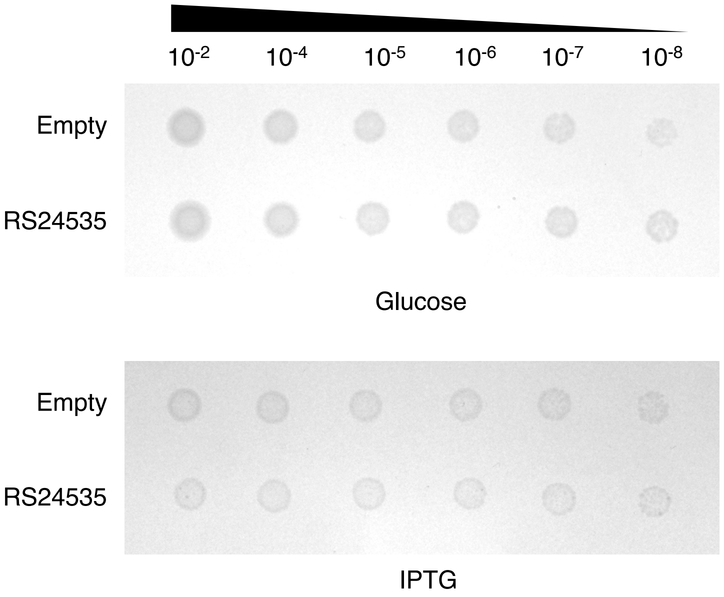

Supplemental Figure 19. The C-terminal region of RS24535, cloned from *A. mediterranei*, did not inhibit *E. coli* growth.

The C-terminal region of RS24535 was cloned into the pET26b plasmid, and serially diluted cultures of the transformed *E. coli* NiCo21(DE3) were spotted onto solid LB media containing 0.2% (w/v) glucose or 0.15 mM IPTG as a repressor or inducer, respectively.

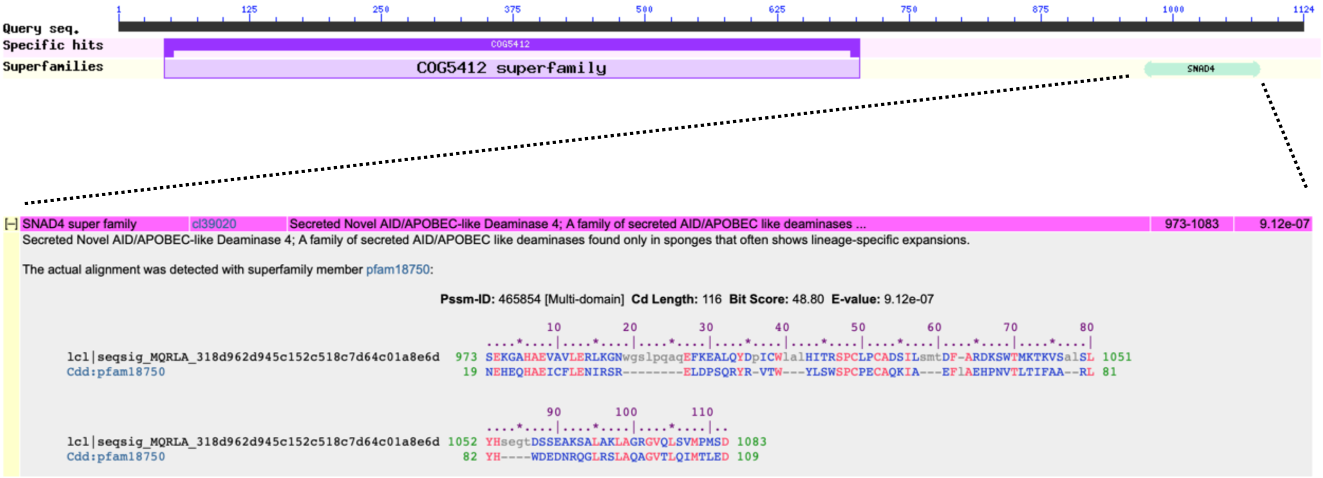

Supplemental Figure 20. Identification of a putative cytidine deaminase domain in RS14790 from *Micromonospora eburnea*.

The amino acid sequence of RS14790 was subjected to a Conserved Domain Search in the NCBI database. This informatic analysis indicated significant homology between a subclass of the AID/APOBEC enzyme family and the C-terminal domain of RS14790.

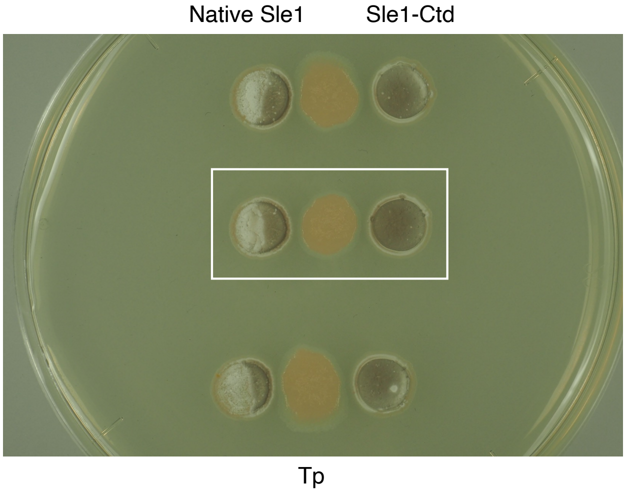

Supplemental Figure 21. Consistent difference in the co-culture phenotypes of the *S. lividans* strains.

The *S. lividans* strains were co-cultured with *T. pulmonis*. The colonies shown in Fig. 5F is indicated by white square.

Supplemental Table 1. Bacterial strains used in this study

Strain	Description	Reference
S. lividans TK23	Parental strain	Lab stock
S. lividans Δ*slpS*	SLP-deficient mutant	Ref. 10
S. lividans Δ*sle2*	Sle2-deficient mutant	This study
S. lividans Δ*sle1/2*	Sle1/2-deficient mutant	This study
S. lividans Δ*sle1/2 attC*::*sle1/2*	Sle1/2-complemented strain	This study
S. lividans Δ*sle1/2 attC*::*sle1(hibit)/sle2*	Sle1(HiBiT)/Sle2-complemented strain	This study
S. lividans attC::P_{tipA}-RS-*msfgfp*	msfGFP-expressing strain	This study
S. lividans Δ*slpS attC*::P_{tipA}-RS-*msfgfp*	msfGFP-expressing strain	This study
S. lividans Δ*sle1/2 attC*::P_{tipA}-RS-*msfgfp*	msfGFP-expressing strain	This study
S. lividans Δ*sle1/2 attC*::*sle1-RS14785-C/sle2*	Sle1 variant-expressing strain	This study
S. lividans Δ*sle1/2 attC*::*sle1-msfgfp/sle2*	Sle1 variant-expressing strain	This study
S. griseus IFO 13350	Competitor strain	Lab stock
T. pulmonis	Competitor strain	Lab stock
E. coli NiCo21(DE3) pCold::*sle2*	His_6-Sle2-expressing strain	This study
E. coli NiCo21(DE3) pET26b	Control strain	This study
E. coli NiCo21(DE3) pET26b::*FLAG-slpT2*	FLAG-SlpT2-expressing strain	This study
E. coli NiCo21(DE3) pET26b::*FLAG-slp4*	FLAG-Slp4-expressing strain	This study
E. coli NiCo21(DE3) pET26b::*FLAG-slp5*	FLAG-Slp5-expressing strain	This study
E. coli NiCo21(DE3) pET26b::*3xFLAG-sle1-N*	FLAG-Sle1-N-expressing strain	This study
E. coli NiCo21(DE3) pET26b::*3xFLAG-sle1-C*	FLAG-Sle1-C-expressing strain	This study
E. coli NiCo21(DE3) pET26b::*sle1-C*	Sle1-C-expressing strain	This study
E. coli NiCo21(DE3) pET26b::*3xFLAG-sle1-C* pBAD::*msfgfp*	Co-expression strain for 3xFLAG-Sle1-C and msfGFP	This study
E. coli NiCo21(DE3) pET26b::*RS24535-C*	RS24535-C-expressing strain	This study
E. coli NiCo21(DE3) pET26b::*RS14790-C*	RS14790-C-expressing strain	This study
E. coli NiCo21(DE3) pET26b pBAD	Control strain	This study
E. coli NiCo21(DE3) pET26b::*RS14790-C* pBAD	RS14790-C-expressing strain	This study
E. coli NiCo21(DE3) pET26b::*RS14790-C* pBAD::*RS14785*	Co-expression strain for RS14790 and RS14785	This study
E. coli NiCo21(DE3) pMAL::*sle1-C*	His_6-MBP-Sle1-C-expressing strain	This study
E. coli NiCo21(DE3) pET15b	Control strain	This study
E. coli NiCo21(DE3) pET15b::*sle1*	Sle1-expressing strain	This study
E. coli NiCo21(DE3) pET15b::*sle2*	Sle2-expressing strain	This study

Supplemental Table 2. Plasmids and primers used in this study

Plasmid	Description
pK18mob::*sle2*-FR12	Gene deletion
pK18mob::*sle1/2*-FR12	Gene deletion
pTYM19t::*sle1/2*	Gene complementation
pTYM19t::*sle1(hibit)/sle2*	Gene complementation
pTUM19t::P_{tipA}-RS-*msfgfp*	Gene complementation
pTYM19t::*sle1-RS14785-C/sle2*	Gene complementation
pTYM19t::*sle1-msfgfp-C/sle2*	Gene complementation
pCold::*sle2*	Protein expression and purification
pET26b::*FLAG-slpT2*	Protein expression
pET26b::*FLAG-slp4*	Protein expression
pET26b::*FLAG-slp5*	Protein expression
pET26b::*3xFLAG-sle1-N*	Protein expression
pET26b::*3xFLAG-sle1-C*	Protein expression
pET26b::*sle1-C*	Protein expression
pET26b::*RS24535-C*	Protein expression
pET26b::*RS14790-C*	Protein expression
pBAD::*msfgfp*	Protein expression
pBAD::*RS14785*	Protein expression
pMAL::*sle1-C*	Protein expression and purification
pET15b::*sle1*	Protein expression
pET15b::*sle2*	Protein expression

Primer	Sequence
Sle2-FR1_Fw	TATGACCATGATTACGAATTGGCACTCGGGGCAGTACTCG
Sle2-FR1_Rv	GAGGCCAAGGAGTCGCCCAG
Sle2-FR2_Fw	CTGGGCGACTCCTTGGCCTC CCCTTCGACAGCGGACGTGT
Sle2-FR2_Rv	ACGACGGCCAGTGCCAAGCTAGGGAATCCGCGGAACTGGC
Sle1/2-FR2_Fw	CTGGGCGACTCCTTGGCCTC TTCTTCTGCTCCGCCACCTC
Sle1/2-FR2_Rv	ACGACGGCCAGTGCCAAGCT TCTCCTCGTCGCACTTGACG
Sle1/2_pTYM19t_Fw	GTGAATTCGAGCTCGGTAC GACGTGCCCACTCCCTCGATCG
Sle1/2_pTYM19t_Rv	ATGACCATGATTACGCCA CTACTCCGCGTCCTCCACCTGGT
GGGGSx2-HiBiT_Fw	GGCGGTGGCGGGTCCGGCGGTGGCGGGTCCGTCTCCGGCTGGCGCCTCTTCAAGAAGATCTCG
GGGGSx2-HiBiT_Rv	CGAGATCTTCTTGAAGAGGCGCCAGCCGGAGACGGACCCGCCACCGCCGGACCCGCCACCGCC
Sle1(HiBiT)_Rv	CCGCCGGACCCGCCACCGCC GAGCGAGCCGAGCTGTTTGGCG
HiBiT-Sle2_Fw	GCCTCTTCAAGAAGATCTCG TGAGCGCCGTGTGCCCCG
msfGFP_pTYM19t_Fw	CTGCTAAGGAGGCAACAAG ATGGTCTCGAAGGGCGAGGAGC
msfGFP_pTYM19t_Rv	ACCATGATTACGCCA GGCCTCTAGTGCCTTCGACGA
Sle2_Fw	TGAGCGCCGTGTGCCCCG
msfGFP_Sle1_Fw	GGCGGTGGCGGGTCCGGCGGTGGCGGGTCC ATGGTCTCGAAGGGCGAGGAGC
msfGFP_Sle1_Rv	CCCGGGGCACACGGCGCTCA CTTGTACAGCTCGTCCATGCCGT
RS14790-C_Sle1_Fw	GGCGGTGGCGGGTCCGGCGGTGGCGGGTCC GCCAACGAACAAGACCACTTCCAA
RS14790-C_Sle1_Rv	CCCGGGGCACACGGCGCTCA GTCGGTGTAGCGCTTCTCCGC
Sle2_pCold_Fw	TACCCTCGAGGGATCC ATGGTGCGCGCGCACGAGGA
Sle2_pCold_Rv	TGCAGGTCGACAAGCTT CTACTCCGCGTCCTCCACCTGG
SlpT2_Fw	ATAAGGGCGGTGGCGGTAGC ATGACCCGTAAGGACCCGGGC
SlpT2_Rv	AGTGCGGCCGCAAGCTT TCAGTCCGTGAACCCGTGGTGG
Slp4_Fw	ATAAGGGCGGTGGCGGTAGC ATGGCCAAGAGCAGCAAGGGC
Slp4_Rv	AGTGCGGCCGCAAGCTT TCAGTGAGGCACCTCCTCGGTGG
Slp5_Fw	ATAAGGGCGGTGGCGGTAGC ATGGTGCGTCCCTCCTTCTCCA
Slp5_Rv	AGTGCGGCCGCAAGCTT TCACAGGCCCGGCACGGG
Sle1-N_Fw	ATAAGGGCGGTGGCGGTAGC ATGTCGGACCGCTTGAACCAGA
Sle1-N_Rv	AGTGCGGCCGCAAGCTT TCA GGCCCACGGCTTGGTCTTGG
Sle1-C_Fw	ATAAGGGCGGTGGCGGTAGC GACGTCCCCGACACCGTGGT
Sle1-C_Rv	GGTGCTCGAGTGCGGCCGCA TCAGAGCGAGCCGAGCTGTTTG
RS24535-C_Fw	ATAAGGGCGGTGGCGGTAGC GTTCCGGACCCGCGCAGCG
RS24535-C_Rv	AGTGCGGCCGCAAGCTT TCACTCTTTCCCGTCGGGGCC
RS14790-C_Fw	ATAAGGGCGGTGGCGGTAGC GCCAACGAACAAGACCACTTCCAA
RS14790-C_Rv	AGTGCGGCCGCAAGCTT CTAGTCGGTGTAGCGCTTCTCCG
RS14785_Fw	AGGAGGAATTAACC A TGAGCAAGCTCATAGATGTCGATCATCT
RS14785_Rv	CAAAACAGCCAAGCTT CTAGGACCCCTCGTGCCCG
Sle1_pET15b_Fw	AGGAGATATACC ATGTCGGACCGCTTGAACCAGACGCA
Sle1_pET15b_Rv	GTTAGCAGCCGGATC TCAGAGCGAGCCGAGCTGTTTGGC
Sle2_pET15b_Fw	AGGAGATATACC ATGGTGCGCGCGCACGAGGA
Sle2_pET15b_Rv	GTTAGCAGCCGGATC CTACTCCGCGTCCTCCACCTGG
Sle1-C_pMAL_Fw	CCAAGCGATCGCCATG GACGTGCCGGATACAGTTGTCC
Sle1-C_pMAL_Rv	TACCGAGCTCGAATTC TCA TAGAGACCCAAGTTGCTTGGCA

Supplemental Notes:

1. Sle1 is less stable in the absence of SLP

To examine the fate of Sle1 in the absence of either the interaction with Sle2 or loading into SLP, we performed western blot analysis using anti-Sle1 antibody for the Δ*slpS*, Δ*sle2*, and Δ*sle1/2* mutants and the parental strain. To our surprise, Sle1 was barely detected in any of the mutants, indicating that mature SLPs and Sle2 could eventually affect the biological stability of Sle1 within the mycelia (Supplemental Fig. 2). Thus, Sle2 may facilitate Sle1 loading into mature SLP, protecting Sle1 from degradation.

2. Insights into the role of Sle2 as a CIS core domain (DUF4157)-containing protein

Although Sle2 may be associated with SLP as a cargo, we assume that this protein might alternatively be dedicated to the mediating role in Sle1 loading into SLP. Given the relatively lower abundance of Sle2 compared to the other SLP proteins in the isolated Slp5 (spike protein) fraction, Sle2 may not be incorporated into fully matured SLP (Supplemental Fig. 1). Considering the potential of the DUF4157 domain to physically interact with various proteins (3), Sle2 may guide Sle1 to SLP through its interaction with SLP proteins, particularly SlpT2, during SLP assembly (Fig. 1C and F). The genetic relationship and potential physical interaction between the effector and the DUF4157 domain-containing protein suggest that these proteins are evolutionary ancestors or descendants of Tme and possibly other putative DUF4157 domain-containing CIS effectors that integrate the DUF4157 and the functional domains within one polypeptide (4,5).

3. Sle1 does not negatively affect protein synthesis in E. coli

We were interested in the effect of Sle1 on protein synthesis activity in which the ribosomes play a pivotal role. To examine this, we purified the recombinant Sle1-C and added it to an *in vitro* protein expression system derived from *E. coli* cells (Supplemental Fig. 12A and B). Expression of the indicator protein msfGFP was not affected by the presence of the purified Sle1-C (Supplemental Fig. 12B). We also constructed a co-expression system for msfGFP and Sle1-C in *E. coli* and analysed *in vivo* protein synthesis activity with or without Sle1-C induction by measuring msfGFP fluorescence as an indicator of protein synthesis. Without Sle1-C induction, the msfGFP fluorescence normalised to cell density was the highest during the exponential growth phase and slowly decreased during the stationary phase (Supplemental Fig. 12C and D). In contrast, under Sle1-C induction condition, the normalised msfGFP fluorescence continued to increase

over time after entering the stationary phase (Supplemental Fig.12C and D). These results suggest that Sle1-C delays *E. coli* growth without inhibiting protein synthesis despite the association of Sle1-C with the ribosome fractions.

4. Probable differential effector loading in SLP and CIS^{Sc}

In *S. coelicolor*, ricin-like proteins (SCO4526-4528) have been proposed as putative effectors loaded by CIS^{Sc}, the *S. coelicolor* counterpart of SLP (6). In contrast, their homologs SLIV_17105 and SLIV_17100 were not enriched in the isolated SLP fractions of *S. lividans*, indicating the absence of these ricin-like proteins from SLPs (Figure 1A). This difference in effector loading may reflect the ecological and/or evolutionary backgrounds of the two species. Although *S. coelicolor* has three ricin-like proteins in the upstream region of the CIS gene cluster, only two are conserved in the SLP gene cluster of *S. lividans*, implying that there has been selective pressure driving the duplication or acquisition of the ricin-like proteins in *S. coelicolor* but not in *S. lividans*. Since the ricin-like proteins have been proposed to mediate cell death in *S. coelicolor*, the partial conservation of these proteins in the SLP gene cluster suggests that SLPs are less related to the CIS-mediated cell death in *S. lividans*. Consistent with this idea, we did not observe any signs of SLP-induced cell death in *S. lividans*, with no consistent proteomic trend indicating global SLP-associated stress responses that must be induced during the cell death (Supplemental Fig. 14). Therefore, SLP and CIS^{Sc} can be considered to be functionally distinct, although their key structural proteins are almost identical and *S. lividans* and *S. coelicolor* have been recognised to be genetically quite similar. The potential difference in the functions of SLP and CIS^{Sc} would result from the differential loading of their cognate effectors, raising intriguing questions as to how similar CIS-related nanostructures recognise and load different effectors. For SLP, Sle2 might be the key for the selective loading of Sle1 since this DUF4157 domain-containing protein has been detected only in the isolated SLP fraction but not in the CIS^{Sc} fraction (6).

Supplemental References:

1. Nagakubo, T. *et al.* Intracellular phage tail-like nanostructures affect susceptibility of *Streptomyces lividans* to osmotic stress. *mSphere* **8**, e0011423 (2023).

2. Gilchrist, C. M. and Chooi, Y-H. clinker & clustermap.js: automatic generation of gene cluster comparison figures. *Bioinformatics* **37**, 2473-2475 (2021).

3. Wood, T. E. *et al.* The *Pseudomonas aeruginosa* T6SS delivers a periplasmic toxin that disrupts bacterial cell morphology. *Cell Rep.* **29**, 187-201 (2019).

4. Geller, A. M. *et al.* The extracellular contractile injection system is enriched in environmental microbes and associates with numerous toxins. *Nat. Commun.* **12**, 3743 (2021).

5. Nagakubo, T., Nishiyama, T., Yamamoto, T., Nomura, N., Toyofuku, M. Contractile injection systems facilitate sporogenic differentiation of *Streptomyces davawensis* through the action of a phage tapemeasure protein-related effector. *Nat. Commun.* **15**, 4442 (2024).

6. Casu, B., Sallman, J. W., Schlimpert, S. and Pilhofer, M. Cytoplasmic contractile injection systems mediate cell death. *Nat. Microbiol.* **8**, 711-726 (2023).
